# Characterisation of a K390R ITK Kinase Dead Transgenic Mouse – Implications for ITK as a Therapeutic Target

**DOI:** 10.1371/journal.pone.0107490

**Published:** 2014-09-24

**Authors:** Angela Deakin, Graham Duddy, Steve Wilson, Steve Harrison, Judi Latcham, Mick Fulleylove, Sylvia Fung, Jason Smith, Mike Pedrick, Tom McKevitt, Leigh Felton, Joanne Morley, Diana Quint, Dilniya Fattah, Brian Hayes, Jade Gough, Roberto Solari

**Affiliations:** 1 Respiratory Therapy Area, GlaxoSmithKline, Stevenage, Herts, United Kingdom; 2 Laboratory Animal Sciences, GlaxoSmithKline, Stevenage, Herts, United Kingdom; 3 Platform Technology and Sciences, GlaxoSmithKline, Stevenage, Herts, United Kingdom; Northwestern University Feinberg School of Medicine, United States of America

## Abstract

Interleukin-2 inducible tyrosine kinase (ITK) is expressed in T cells and plays a critical role in signalling through the T cell receptor. Evidence, mainly from knockout mice, has suggested that ITK plays a particularly important function in Th2 cells and this has prompted significant efforts to discover ITK inhibitors for the treatment of allergic disease. However, ITK is known to have functions outside of its kinase domain and in general kinase knockouts are often not good models for the behaviour of small molecule inhibitors. Consequently we have developed a transgenic mouse where the wild type *Itk* allele has been replaced by a kinase dead *Itk* allele containing an inactivating K390R point mutation (*Itk*-KD mice). We have characterised the immune phenotype of these naive mice and their responses to airway inflammation. Unlike *Itk* knockout (*Itk^−/−^*) mice, T-cells from *Itk*-KD mice can polymerise actin in response to CD3 activation. The lymph nodes from *Itk*-KD mice showed more prominent germinal centres than wild type mice and serum antibody levels were significantly abnormal. Unlike the *Itk^−/−^*, γδ T cells in the spleens of the *Itk*-KD mice had an impaired ability to secrete Th2 cytokines in response to anti-CD3 stimulation whilst the expression of ICOS was not significantly different to wild type. However ICOS expression is markedly increased on αβCD3^+^ cells from the spleens of naïve *Itk*-KD compared to WT mice. The *Itk*-KD mice were largely protected from inflammatory symptoms in an Ovalbumin model of airway inflammation. Consequently, our studies have revealed many similarities but some differences between *Itk^−/−^*and *Itk*-KD transgenic mice. The abnormal antibody response and enhanced ICOS expression on CD3+ cells has implications for the consideration of ITK as a therapeutic target.

## Introduction

Interleukin-2 inducible tyrosine kinase (ITK) is a member of the Tec family of non-receptor kinases, which include Tec, Btk, Rlk/Txk and Bmx. ITK is expressed in T lymphocytes, where it plays a pivotal role in the signalling cascade down-stream of the T cell receptor (TCR). ITK is present within the cytoplasm of resting T cells, but following activation of the TCR, becomes localised to the plasma membrane via its pleckstrin homology domain binding to phosphatidylinositol (3,4,5) triphosphate (PIP_3_), and binds with other signalling molecules including phosphorylated LAT and SLP76 to form a signalsome complex, reviewed by [1]. ITK is subsequently activated by Lck which phosphorylates tyrosine 512 in the conserved activation loop within the kinase domain [2]. The best characterised substrate for ITK is PLCγ1, which it phosphorylates at Tyr 775 and 783. Activated PLCγ1 subsequently cleaves phosphatidylinositol-4-5-biphosphate (PIP_2_) to generate the second messengers inositol 1,4,5 triphosphate (IP_3_) and diacylglycerol (DAG) which set in train a well described series of events leading to T-cell activation. ITK is also expressed in NK cells where it plays a role in the response the FcγRIIIa activation [3], and in mast cells, where it is activated following cross-linking of FcεRI but has differential effect on mast cell responses [4].

In addition to its kinase-dependent action, ITK may play an important role as an adaptor protein within the signalsome complex, via interactions with its SH2 and SH3 domains. For example, the SH2 domain of ITK is essential for the binding to LAT, whereas a kinase dead mutant (K390R) co-immunoprecipitated with LAT [5]. ITK has a potential role as a scaffold protein, which helps regulate the localisation of Vav, the guanine nucleotide exchange factor, to the signalsome, where it plays an important role in the regulation of Rho family GTPases [6]. Following activation via the TCR, distinct clusters of signalling molecules localise to the T cell-antigen presenting cell (APC) contact site. This process is critical for activation of T cells through the TCR-CD3 complex and is dependent on the reorganisation and polymerisation of the actin cytoskeleton [7], [8]. Kinase dead ITK failed to disrupt cell contact and polarisation to APC [9] and studies have demonstrated that ITK plays role in actin-polymerisation in response to TCR-CD3 activation, which is independent of its kinase activity [6], [10]. Indeed, so important is the scaffold function of ITK that knocking it out results in the spatiotemporal disruption of 14 of the 16 components of the TCR signalling complex at the interface with the APC [11].

There are several published studies in *Itk*-knockout (*Itk^−/−^*) mice showing that ITK plays an important role in the development, differentiation and activation of T-lymphocytes, in particular Th2 cells. The available evidence suggests ITK is an attractive target for allergic diseases such as asthma and this has prompted us [12] and numerous other pharmaceutical companies to generate inhibitors of this kinase [13]. However, despite substantial effort only one compound (JTE-051, Japan Tobacco) has entered the clinic to date, and it is unknown if this is due to lack of efficacy, poor selectivity or toxicology. As described above, ITK has both kinase-dependent and independent functions, and complete knockouts are unable to distinguish these two functions. A transgenic mouse where the kinase domain was replaced by green-fluorescent protein has been described and showed differential responses to Th2 cytokine production and chemokine mediated migration [14]. However it is quite clear that kinase knockouts and chemical inhibitors can give quite different results [15]. Consequently to support the validation of ITK as a drug target we have generated an ITK kinase dead mouse by replacing the wild type allele with an *Itk* allele carrying a K390R mutation which is known to have no kinase activity [2], [5]. Not only does such a model retain the scaffold function of ITK it is also a model which more closely mimics the actions of a small molecule kinase inhibitor than an ITK null mouse. We have used this genetically modified ITK kinase dead mouse (*Itk*-KD) to further our understanding of the consequences of inhibiting ITK with small molecule kinase inhibitors.

## Materials and Methods

### The Itk targeting strategy

A targeting strategy was devised to introduce the point mutation K390R into exon 12 of the *Itk* gene by homologous recombination in ES cells. 5′ & 3′ homology arms (approx. 3.5 & 2.8 kb respectively) flanking exon 12 were generated using Phusion High-Fidelity DNA Polymerase (New England BioLabs) on a BALB/c genomic DNA template. Similarly a ∼0.6 kb fragment carrying exon 12 lying between these two homology arms was isolated and subjected to site-directed mutagenesis with the QuickChangeII site-directed mutagenesis kit (Stratagene) to introduce the appropriate point mutation (A to G mutation at n1169 of the cDNA sequence). The 5′ & 3′ homology arms and the mutated exon 12 fragments were subcloned into a parental targeting vector to achieve the positioning of the loxP & FRT sites and the neo cassette indicated in the schematic (Figure S1 in File S1). Gene targeting was performed in de novo generated BALB/c ES cells. The targeting construct was linearised and electroporated into ES cells according to standard methods. ES cells correctly targeted at the 3′ end were identified by Southern blot analysis using a PCR-derived external probe. Correct gene targeting at the 5′ end and the presence of the appropriate point mutation was confirmed by sequencing of a ∼6 kb PCR product. The latter was generated by high-fidelity PCR of ES cell clone-derived genomic DNA using primers spanning the 5′ homology arm (data not shown). Note that an additional loxP site was simultaneously introduced into intron 11, but was not used in subsequent model generation. Targeted ES cell clones were injected into C57Bl6/J-derived blastocysts, and resultant male chimaeras were crossed with BALB/c females to produce mice heterozygous for the *Itk* primary targeted allele. These were subsequently bred to a germline Flp-deleter strain resulting in mice heterozygous for the *Itk* knock in allele (*Itk* ^K390R/+^). After an expansion breed to BALB/c study populations were produced by intercrossing sufficient heterozygous pairs to produce mice homozygous for the *Itk* knock in allele (*Itk* ^K390R/K390R^), hereafter called *Itk*-KD, and wild type litter mates. Expression of mRNA from the mutant *Itk* allele was confirmed by sequencing of RT-PCR products derived from spleen RNA of mice heterozygous for the targeted allele (data not shown). Within each experiment age-matched WT and *Itk*-KD mice were used.

All animal studies were reviewed and approved by the local ethics committee; the GSK Policy on the Care, Welfare and Treatment of Animals Committee, and carried out in accordance with UK Animals (Scientific Procedures) Act 1986.

### Phenotyping of Itk-KD mice

Blood samples from 5 male and 5 female mice for WT and *Itk*-KD genotypes were drawn into heparinised tubes. Full differential blood counts were analysed using Advia 120. Further analysis of lymphocyte populations was carried out by flow cytometry, and the plasma stored for subsequent analysis of antibody isotypes (as described below). Mice were culled with an overdose of pentabarbitone, and perfused with 4% paraformaldehyde. Major organs were removed and analysed by histology. Tissues were processed through graded alcohols and xylene into paraffin wax using an enclosed tissue processor (Sakura VIP6). The tissues were then embedded in paraffin wax, sections cut at 4 µm dried and stained with Haematoxylin and Eosin followed by histopathological screening.

### Immunoprecipitation and Western blot for ITK

Splenocytes were gently squeezed from the spleen through a 40 µm cell strainer using a syringe plunger. Contaminating red blood cells were lysed on ice for 1 min (0.15M NH_4_Cl, 0.01M KHCO_3_, 0.001M EDTA, pH7.4), the cells washed and re-suspended in complete media (RPMI 1640, 10% heat inactivated fetal calf serum, 2 mM L-glutamine, 100 U/ml penicillin, 100 µg/ml streptomycin and 50 µM β-mercaptoethanol). Lymphocyte suspensions were made from the lymph nodes by dissociation between two glass slides in complete assay media.

CD4^+^ cells were isolated by positive selection using anti-CD4 (LT34)-coated microbeads and B cells using anti-CD45R (B220)-coated microbeads (Miltenyi Biotec) from pooled lymph node and splenocyte cell suspensions. The resulting CD4^+^ preparations were >84% pure, and the B cell preparation >91% pure. Cells were lysed with ice cold lysis buffer (50 mM Tris-Cl pH 7.6, 150 mM NaCl, 1% (w/v) n-Octyl-β-D glucoside, 50 mM NaF, 1 mM Na_3_VO_4_ (all from Sigma), 1% Triton X-100 (Thermo Scientific), containing protease inhibitor cocktail (Roche)). Lysates (following pre-clearing with protein-G agarose), were incubated with mouse monoclonal anti-ITK (2F12, BD Biosciences) followed by protein-G agarose to immunoprecipitate ITK. Washed immune complexes were heated (70°C, 10 min) in ×4 Nu-PAGE sample loading buffer with reducing agent (Invitrogen) and separated on a 4–12% Bis-Tris Gel (Invitrogen). Proteins were transferred to PVDF membrane (Novex) and blocked for 30 min at room temperature (3% non-fat milk TBS (Sigma)). Membranes were incubated (overnight at 4°C) with a polyclonal rabbit anti-ITK (Millipore; 1 µg/ml in 3% non-fat milk TBS). Following extensive washing (0.05% Tween-20 PBS), membranes were incubated (30 min, room temperature) with HRP-coupled goat anti-rabbit IgG (cell signalling, 1∶3000 in 3% non-fat milk TBS). Membranes were washed again and blots developed using SuperSignal West Femto (Thermo Scientific) and imaged on high performance chemiluminescent film (Hyperfilm ECL, Amersham).

### Anti-CD3 induced actin polymerisation in CD4^+^ cells

CD4^+^ cells were isolated from splenocytes of naїve mice by negative selection using MCD43 columns (R&D Systems) to give 93% CD4^+^ cells with >97% viability. Cells were washed in serum free media (RPMI 1640, 2 mM L-glutamine, 100 U/ml penicillin, 100 µg/ml streptomycin and 50 µM β-mercaptoethanol) and re-suspended at 1×10^7^/ml in serum free media. Using a volume of 0.2 ml per test, cells were allowed to rest for 30 min, (37°C, 5% CO_2_), before activation with anti-CD3 coated beads (Miltenyi) at a ratio of 1 bead per cell. The cell suspensions were centrifuged (150 g, 1 min) to ensure contact between the beads and cells, and then the complexes re-suspended by gentle pipetting, and incubated (37°C) for a further 10 min. Cells were fixed by adding an equal volume of Cytofix (BD Biosciences) and incubated for a further 10 min (room temperature). Cells were centrifuged (350 g, 5 min), washed in stain buffer (2% heat inactivated FCS, 0.05% sodium azide in PBS) and cells pelleted (350 g, 5 min). Cells were permeabilised (1 ml per test of perm/wash buffer, BD Biosciences) for 15 min (room temp), centrifuged (350 g, 5 min), and the cells re-suspended in Alexa-488 phalloidin (Invitrogen, diluted 1∶40 in perm/wash buffer). Following incubation (20 min), cells were washed (twice in perm/wash buffer) and re-suspended in PBS. Cell: bead complexes were examined on a Leica TCS SP5 microscope using a 20× objective lens. Single confocal sections of intact cells (through the centre of the cells) were collected into two channels of the microscope (Ch1 488ex 530em, Ch2 transmission image). Four fields per slide were captured. Leica QWin image analyser was used to quantify the staining; the beads are detected by grey thresholding and the resulting binary image is enlarged by 1.5× bead diameter. The intensity of the actin staining in the vicinity of the beads over this 1.5× bead diameter was then measured, and the number of beads counted.

### In vitro activation of splenocytes with anti-CD3

Splenocytes (1×10^6^) were incubated with hamster anti-CD3 (1 µg/ml; clone 145-2C11, low endotoxin, azide free, BD Bioscience) in complete media (200 µl/well). Following incubation (72 h, 37°C, 5% CO2), supernatants were removed and assayed for cytokine release by immunoassay from Meso Scale Discovery (MSD) according to their protocol.

### Flow cytometry

Treg cells were analysed using a kit from Biolegend (according to manufacturers' protocol). For cell surface markers in cell suspensions, washes and incubation steps were carried out in stain buffer (1–2% HI FCS, 0.05% sodium azide in PBS), for BAL cells DNaseI (25 µg/ml; Roche) was added to the buffer. Cells were incubated (5 min) with Fc block (BD Biosciences anti-CD16/32; clone 2.4 G2) at a ratio of 1 µg: million in approximately 100 µl. Cells were stained with the following antibodies; CD4-PerCp-Cy5.5 (RM4-5), CD62L-APC (MEL-14), CD44-PE (IM7), CD3e-APC-Cy7 (145-2C11), CD3-FITC (17A2), CD8a-PE-Cy7 (53-6.7), CD45-APC-Cy7 (30-F11), CD19-APC (ID3), TCRγδ-PE (GL3), rat IgG1–FITC (R3-34), rat IgG1-APC (R3-34), rat IgG2b-PE (A95-1), rat IgG2a-APC (R35-95) hamster IgG2k-PE (B81-3) all from BD Biosciences. T1/ST2-FITC (DJ8) from MD Biosciences, TIM3-PE (8B.2C12) from eBioscience, and CD278 (ICOS)-APC (398.4A) or hamster IgG-APC (HTK888) from Biolegend. For whole blood, 20 µl blood was mixed with Fc block (10 µl at 50 µg/ml). Following incubation (10 min), antibody cocktail was added and incubated for a further 20 min). IOtest 3 (200 µl; Beckman Coulter) was added to each well, mixed and incubated for a further 15 min, and the cells thoroughly washed in stain buffer (containing DNase). DAPI (4′, 6-diamidino-2-phenylindole, Sigma) at 1 µg/ml was typically added to stain dead cells and samples analysed using BD FACS Canto II using FACS Diva v6 software. Multicolour compensation was performed using anti-rat Ig κ or anti-rat/hamster Ig κ CompBead (BD Bioscience). Flow-count flouorospheres (Beckman Coulter) were used to enable absolute cell count determination in the BAL and lymph node.

### Intracellular cytokine measurements

Splenocytes (1×10^6^) were incubated with PMA (50 ng/ml; Sigma)) and ionmycin (1 µg/ml; Sigma) in complete media (200 µl/well) containing Golgi stop (1∶1500 dilution; BD BioSciences). Following an incubation (5 h, 37°C, 5% CO_2_), cells were centrifuged (350 g, 5 min) and re-suspended in Fixable viability dye eFluor 450 (1∶1000 in PBS; eBioscience). Following 30 min incubation (4°C), cells were centrifuged (350 g, 5 min), supernatant aspirated, cells re-suspended in Cytofix (BD Biosciences), and incubated for a further 15 min (room temperature). Cells were washed (x2 stain buffer), before re-suspending in perm/wash buffer (BD Biosciences) and incubated for 15 min (room temperature). Cells were pelleted (350 g, 5 min) and re-suspended in 50 µl perm/wash buffer containing 20 µl staining cocktail (Th1/Th2/Th17 kit; BD Biosciences) or negative controls. Following a further incubation (30 min, room temperature), cells were washed (x2 stain buffer), re-suspended in PBS and analysed on BD FACS Canto II as above.

### Detection of antibody isotypes

IgA, IgG1, IgG2a, IgG2b, IgG3 and IgM were measured using a kit (Mouse Isotyping Panel 1 kit) from MSD according to their protocol. Plasma samples were diluted 1∶1000, to 1∶125,000 to ensure samples reading within the linear range of the assay for each antibody.

To measure total IgE, standard 96-well MSD plates were coated (overnight, 4°C) with anti-IgE (2 µg/ml, PBS, BD). All further incubations were carried out at room temperature on an orbital shaker. Unbound antibody was removed by washing (0.05% Tween 20 in PBS), and non-specific binding blocked (1% BSA/PBS, 200 µl/well) for 1 h. The plate was washed, 25 µl standard (14–10,000 ng/ml, purified mouse IgE, BD) or sample (diluted 1∶10) added and incubated for 2 h. Unbound antibody was removed by washing and 25 µl biotinylated anti-mouse IgE (1 µg/ml; BD) pipetted per well. Following 1 h incubation, the plates were washed and 25 µl streptavidin Sulfo-Tag (1 µg/ml; MSD) added for a further 1 h. Plates were then washed, 150 µl Read buffer (T- x2; MSD) added per well and the samples analysed on SI6000 (MSD). OVA-specific IgE was measured by ELISA (AbD Serotec) according to their protocol.

### Purification of gamma delta T cells

CD3^+^ γδ^+^ T cells were isolated from splenocytes using a two-step procedure (TCRγ/δ T cell isolation kit; Miltenyi Biotech). Non-T cells were magnetically labelled with a cocktail of CD45R (B220) and CD11b antibodies conjugated to micobeads. The TCRγ/δ T cells are labelled with anti-TCR γ/δ-biotin. The magnetically labelled unwanted cells are depleted. The TCRγ/δ T cells are then indirectly magnetically labelled with anti-biotin microbeads and isolated by positive selection from the pre-enriched T cell fraction.

### Preparation of single cell suspension from the lungs

Lungs were placed in complete media, the trachea and bronchus carefully removed. The tissue was chopped with scissors and the cell suspension gently squeezed through a 40 µm cell strainer using a syringe plunger. Cells were washed and re-suspended in staining buffer (2% heat inactivated FCS, 0.05% sodium azide in PBS).

### Ovalbumin (OVA) model of airway inflammation

Female mice (approx 8–10 weeks old) were sensitised to OVA (10 µg; Sigma) emulsified in aluminium hydroxide (2 mg; Sigma) in 0.2 ml saline, given by intraperitoneal (i.p.) injection on day 0 and 14. On day 24, 25 and 26, mice were challenged by the intranasal route (under isoflurane-induced general anaesthesia) with a 50 µl volume of either OVA (1 mg/ml) or saline.

Airway hyper-reactivity (AHR) to 5-hydroxytryptamine (5-HT) was assessed on day 27 using whole body plethysmography (Buxco). This technique measures changes in air pressure as a result of inspiration and expiration. In these experiments, enhanced pause (Penh), a measurement of changes in expiratory effort, was used as an indicator of airway obstruction. Following determination of lung airway function at baseline, mice were exposed to nebulised vehicle (water), and then increasing concentrations (1–10 mg/ml) of 5-HT.

Animals were culled on day 28 (48 h after last challenge with OVA), a terminal blood sample was taken by severing the carotid artery. The trachea was cannulated and the lungs were lavaged by flushing twice with a 1 ml volume of BAL fluid (PBS, 0.1%BSA, 10 mM EDTA). Recovered cells (approx 0.8 ml) were kept on ice and a differential cell count determined by flow cytometry.

At necropsy, the thoracic cavity was opened and the trachea was clamped and cannulated. The lungs were manually instilled with 1 ml of 10% neutral buffered paraformaldehyde using slow gentle pressure on a 1 ml syringe. Once inflated, the lungs were removed from the thoracic cavity, held by the trachea for about 30 seconds, the tracheal clamp removed, and the lungs post-fixed in 10% neutral buffered paraformaldehyde for a minimum of 24 hours.

Following fixation the lungs were processed to paraffin overnight using a VIP automated tissue processor (Bayer). Serial 5 µm sections were cut through each block. The first section from each block was stained with Haematoxylin and Eosin (H&E) using a standard protocol, to demonstrate general lung morphology and inflammation (H&E stains supplied by Pioneer Research Chemicals, Colchester, Essex, UK). Subsequent sections were used for immunohistochemistry to demonstrate the presence of eosinophils (using a polyclonal goat anti-mouse eosinophil major basic protein (eMBP) antibody (sc-33938, Santa Cruz Biotechnology) or T-cells (using a rabbit monoclonal anti-CD3-human antibody, (clone SP7, Thermo Scientific) that cross-reacts with mouse. All immunohistochemistry was performed using a Leica bond automated immunostainer. Briefly, deparaffinised slides were pre-treated with on-board HIER at pH6 (eMBP) or pH8 (CD3) prior to incubation with the primary antibodies for 16 minutes. The secondary antibodies, incubated for 8 minutes, were a biotinylated rabbit anti-goat secondary (Vector Laboratories) for the eMBP, and a RTU goat anti-rabbit pre-adsorbed against mouse for CD3 (Menarini Diagnostics), respectively. Positively stained eosinophils and T-cells were detected using strepavidin-HRP conjugate and 3,3′-diaminobenzidine tetrahydrochloride (DAB). Sections were counterstained using haematoxylin. All stained sections were coverslipped and mounted in DPX mountant before being digitally imaged using a Nanozoomer digital imaging system (Hamamatsu).

The spleen and lymph nodes were also removed, and single cell preparations prepared. Cells (1×10^6^ for splenocytes and 5×10^5^ for lymph nodes) were activated with OVA (1,10 and 100 µg/ml) in a volume of 200 µl in U-bottom plates. Following 72 h incubation, supernatants were removed and cytokines measured by immunoassay (MSD) according to manufacturers' protocol. Intracellular cytokine staining in response to PMA/ionomycin was also measured as described above.

A separate study was carried out to measure cytokines in the BAL. These animals were sensitised and challenged as described above, with exception that the animals were culled 4 h post the final OVA challenge (the optimal time for measuring cytokines). Serial tail bleeds (50 µl) were taken (prior to the first OVA/alum injection on day 0, days 14, 21 and a terminal bleed on day 28) to measure antibody levels in response to immune stimulation.

### Statistical analysis

Data were subjected to ANOVA analysis to determine if there is any effect of treatment (P<0.05). If ANOVA P<0.05 then a post hoc test was performed; either Fisher's least significant difference (LSD) with Hochberg correction or Tukey correction (with P value, *<0.05, **P<0.01). If data were not normally distributed (unequal residuals) then a log10 transformation was applied.

## Results

### Generation and characterisation of the Itk-K390R Kinase Dead transgenic mouse

**Figure 1:**
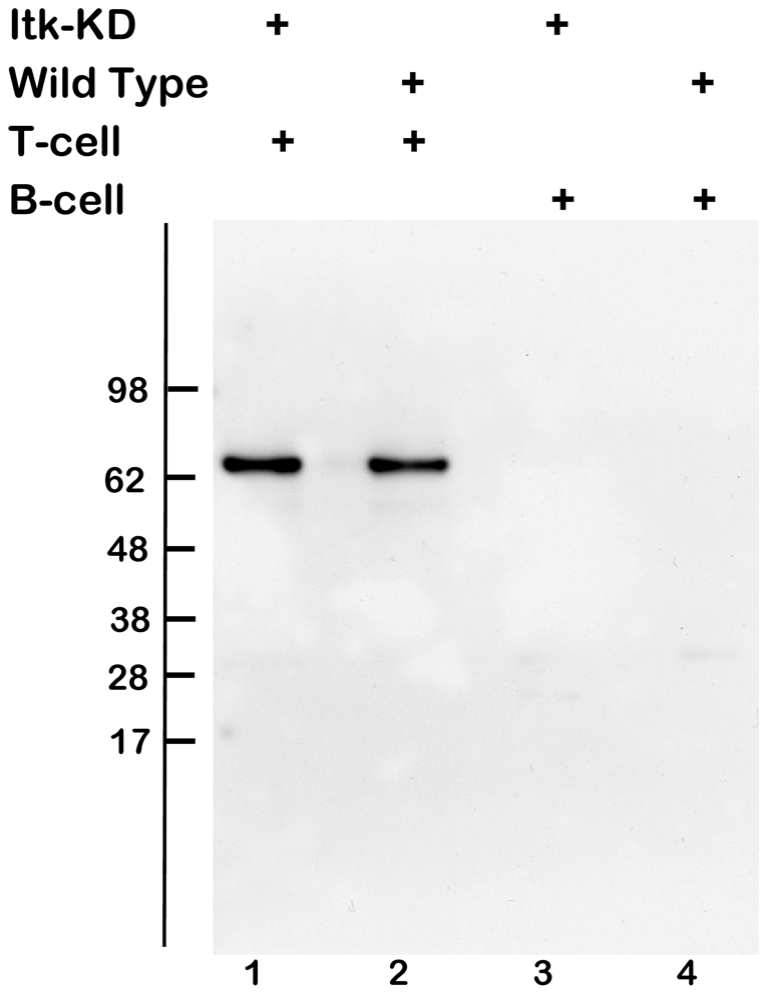
ITK(K390R) is expressed in *Itk*-KD T-cells. Western Blot (WB) following immune precipitation (IP) of lysates from purified T & B cells (combined lymph node & spleens) of Wild Type (WT) and *Itk*-KD mice. Monoclonal antibody anti-ITK (clone 2F12) was used for the IP and polyclonal rabbit anti-ITK for WB.

The K390R mutation in ITK is known to generate an inactive kinase [5] consequently we devised a strategy to introduce this point mutation into exon 12 of the *Itk* gene by homologous recombination (Figure S1 in File S1). We generated *Itk* K390R transgenic BALB/c mice (*Itk*-KD) as described in Materials and Methods and confirmed correct gene targeting by DNA sequencing. In order to confirm ITK was expressed in T cells in *Itk*-KD mice, CD4^+^ cells were isolated from pooled lymph nodes and spleen, and cell lysates immunoprecipitated with an anti-ITK monoclonal antibody followed by Western blotting with an anti-ITK rabbit polyclonal antibody. Figure 1 demonstrates expression of ITK protein in CD4^+^ cells from *Itk*-KD mice that migrates with an identical molecular mass to ITK in WT mice. As a specificity control, lysates of B cells show no immunoreactivity.

**Figure 2:**
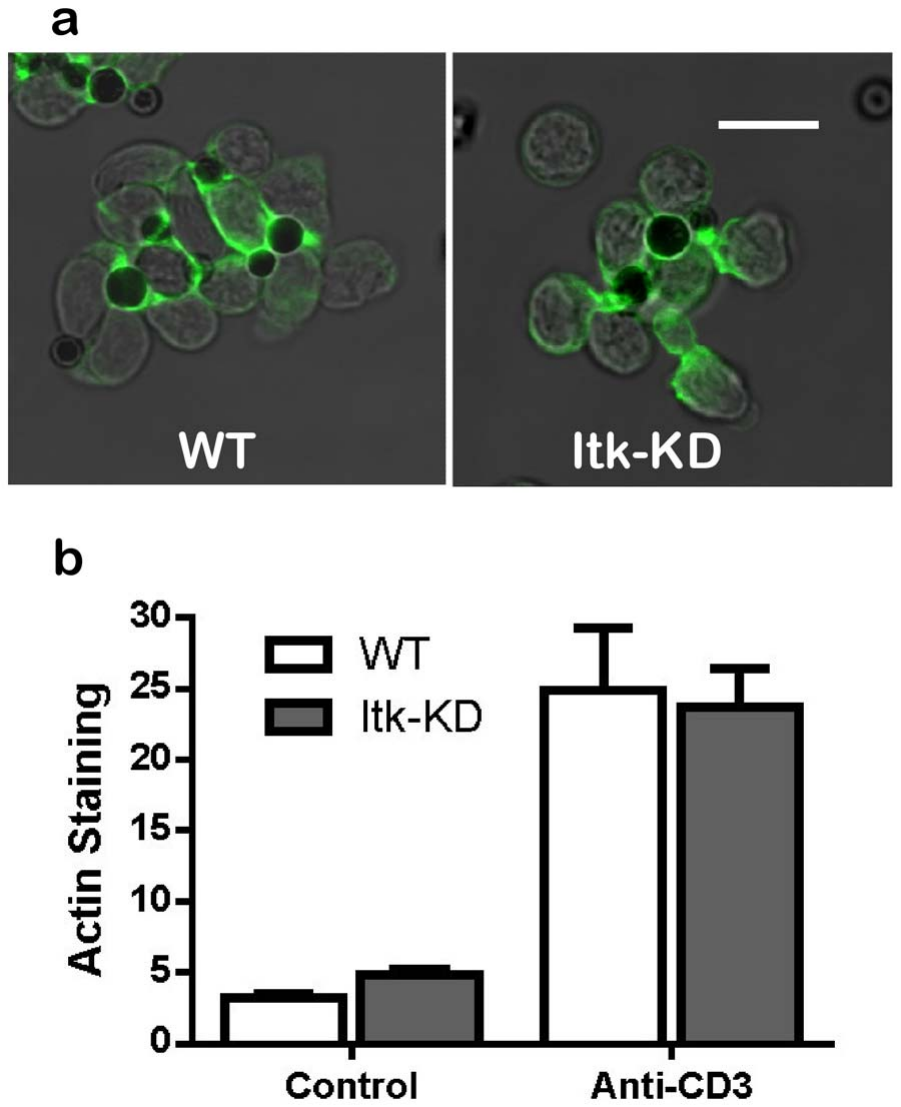
ITK(K390R) retains scaffold function. CD4^+^ cells were isolated from the spleens of naïve and *Itk*-KD mice and incubated with anti-CD3 coated beads. Capping of actin was revealed by staining with Alexa-488-phalloidin (a). The images are single confocal sections of 0.4 µm thickness and the scale bar  = 10 µm. (b) Image analysis of actin capping. Polymerised actin surrounding the anti-CD3 coated beads was quantified as described in Materials and Methods. Data represents the means +/- S.E.M. from four determinations.

Actin polymerisation following activation with anti-CD3 is reported to be dependent on ITK expression but independent of its kinase activity [6], [10], therefore we used an assay to demonstrate that the ITK-K390R protein retains the ability to induce actin polymerisation. Capping of actin, as measured by staining with Alexa-488-phalloidin in response to anti-CD3 coated beads was equivalent in splenic CD4^+^ cells from *Itk*-KD and WT mice (Figure 2).

**Figure 3:**
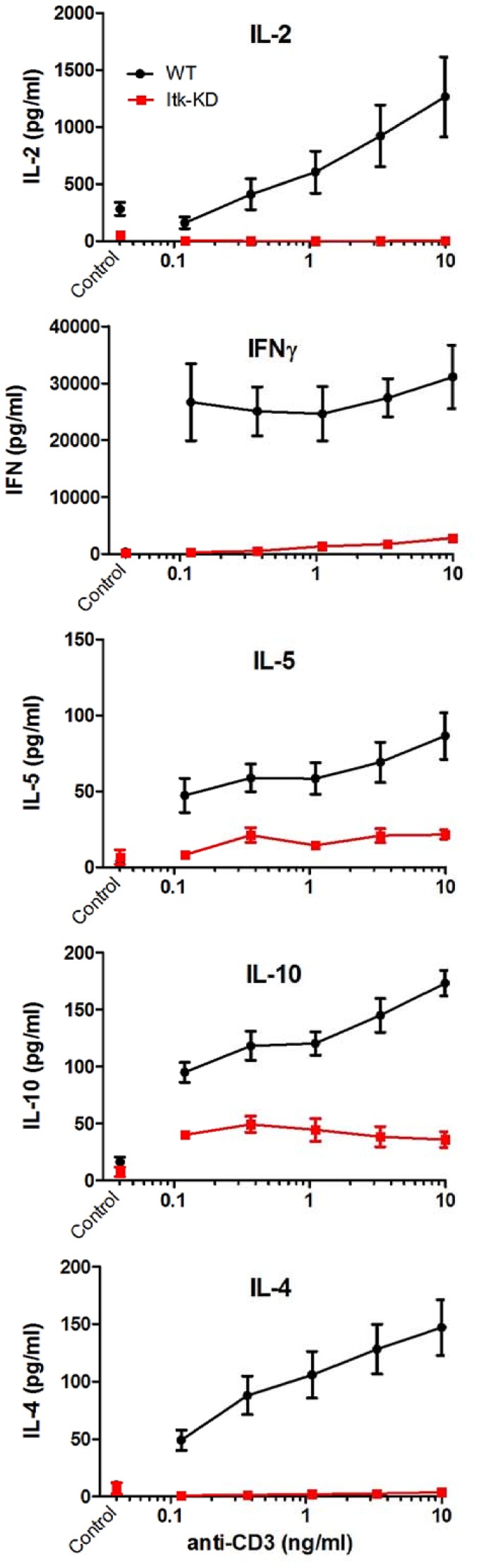
Splenocytes from naïve *Itk*-KD mice fail to secrete cytokines. Splenocytes from WT or *Itk*-KD mice were activated *in vitro* with increasing concentrations of soluble anti-CD3 for 72 h. Cell culture supernatants were collected and secreted cytokines quantified by MSD assays. Results are shown as mean +/− S.E.M. (n = 6 mice).

Cytokine release following activation of T lymphocytes with anti-CD3 is widely reported to be dependent on ITK kinase activity [16]–[19]. Following activation with soluble anti-CD3, splenocytes from WT mice produce IFNγ, IL-2, IL-4, IL-5 and IL-10. By contrast, production of these cytokines is markedly reduced in splenocytes from *Itk*-KD mice, consistent with them lacking ITK kinase activity (Figure 3). Therefore, we have generated transgenic BALB/c mice where the wild type *Itk* gene is replaced with a kinase dead mutant that is expressed at similar levels to the wild type protein that retains its scaffold function but is defective in T cell activation.

### Phenotype of Itk-KD mice

*Itk*-KD mice are viable and fertile and no adverse effect of the *Itk*-KD K390R mutation was observed on weight gain or behaviour. No gross effects on animal health were noted in animals up to 1 year of age. No abnormalities were detected in histopathology screening of kidney, liver, lungs or heart; similarly, no abnormalities were detected in the mesenteric lymph node, mandibular lymph node or thymus in mice at 9 weeks of age. However prominent germinal centres were observed in all male *Itk*-KD and 2 of 5 female *Itk*-KD mice (Figure S2 in File S1). These were considered to be within the normal limits for an active spleen, but no such changes were ever observed in the WT BALB/c mice.

### Comparison of leukocyte populations in the naïve Itk-KD mice to WT

No significant difference was seen between the absolute neutrophil, monocyte or eosinophil cell counts in the blood of naïve WT or *Itk*-KD mice (Figure S3A in File S1). However, there was a significant decrease in the absolute lymphocyte counts. Further analysis by flow cytometry demonstrated a significant decrease in the proportion of CD3^+^ cells in the blood, but no significant effect on B cells, CD4^+^ or CD8^+^ cells (Figure S3B in File S1). Analysis of the cell populations in the spleen by flow cytometry indicated that the *Itk*-KD mice had equivalent numbers of CD4^+^ cells as WT naïve controls (2.85±0.26×10^7^ cells ±S.E.M. and 2.36±0.31×10^7^ cells ±S.E.M. respectively, n = 6). In a separate experiment a similar proportion of CD25^+^CD4^+^FOXP3^+^ T Regulatory cells were measured in *Itk*-KD spleen (6.4±0.4%±S.E.M., n = 6) compared to WT mice (5.6±0.7%±S.E.M., n = 6) when calculated as a percentage of total CD4^+^ cells.

**Figure 4:**
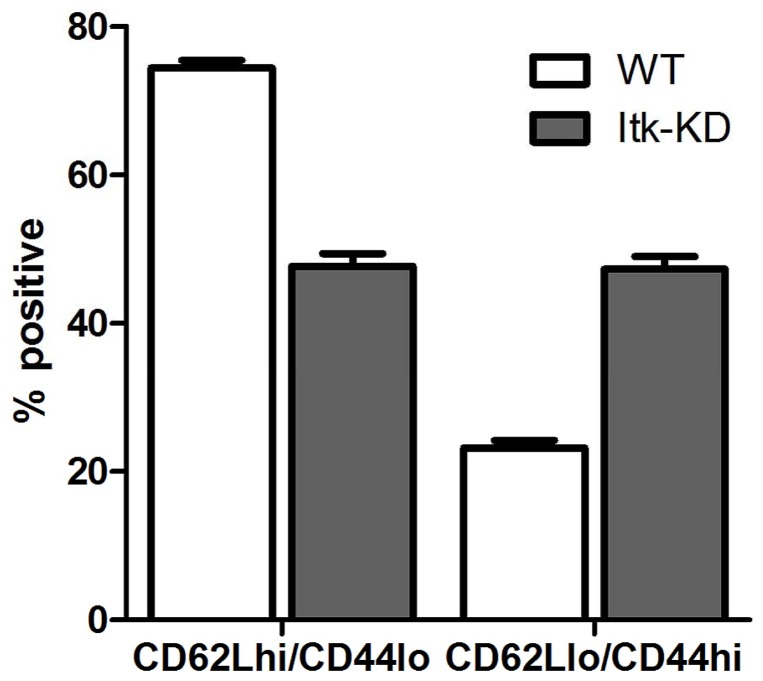
Increased memory markers on CD4^+^ cells. There is an increase in CD4 positive cells that express memory markers CD62L^lo^CD44^hi^ in the spleen of naïve *Itk*-KD mice compared to WT controls. Results are shown as the mean +/− S.E.M. n = 6 mice).

**Figure 5:**
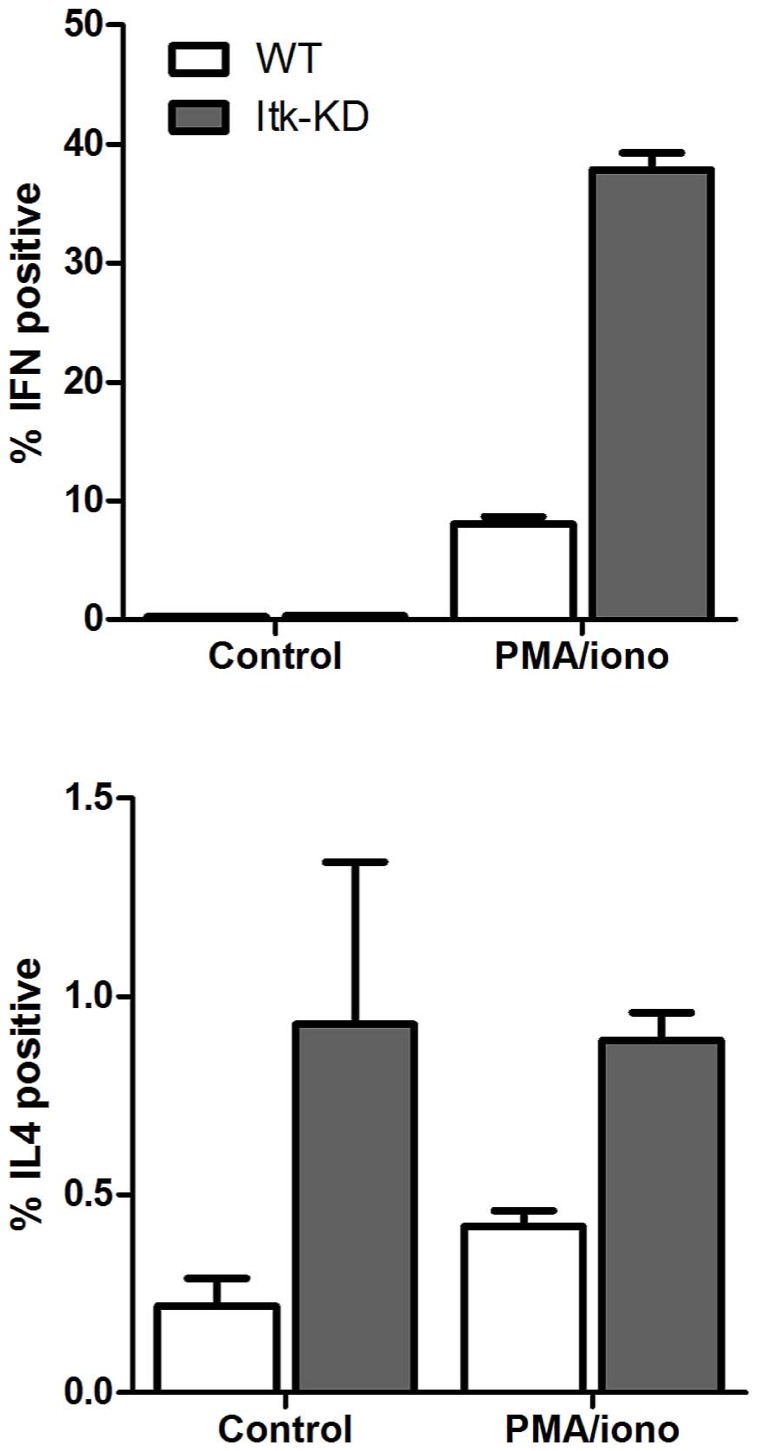
Cytokine secretion by splenocytes following PMA/ionomycin stimulation. Splenocytes from WT or *Itk*-KD mice were activated *in vitro* with PMA (50 ng/ml)/ionomycin (1 µg/ml) for 5 h and intracellular IFNγ and IL-4 determined by flow cytometry. There is an increase in the proportion of CD4^+^ cells from the spleens of *Itk*-KD mice which stain positively for IFNγ and IL-4 than WT. Results represent the mean +/− S.E.M. n = 6 mice.

Further investigation of the CD4^+^ cells demonstrated an increase in the proportion of cells expressing memory markers (CD62L^low^ CD44^high^) in the spleen of *Itk*-KD compared to WT mice (Figure 4). In addition, on activation of splenocytes with PMA and ionomycin, which by-passes ITK, there was an increase in the proportion of CD4^+^ cells that are capable of producing IFNγ in *Itk*-KD compared to WT. Similarly, *Itk*-KD splenocytes produce increased IL-4 in response to PMA/ionomycin compared to WT although the percentage of CD4^+^ cells positive for intracellular IL-4 was low (Figure 5). This suggests that the diminished IFNγ and IL-4 release following activation of *Itk*-KD splenocytes (Figure 3) is not due to a decrease in the presence of CD4^+^ cells capable of producing cytokines but a failure in these cells to respond to anti-CD3 activation.

### Naïve Itk-KD mice show elevated levels of antibody

**Figure 6:**
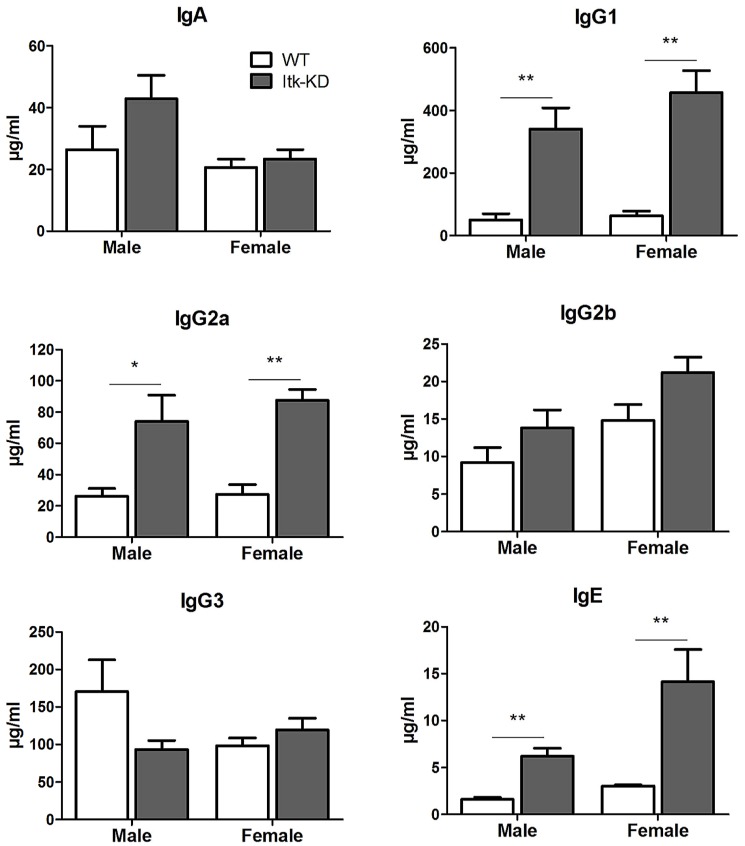
Antibody levels in the plasma of naïve mice. *Itk*-KD mice exhibit increased IgE, IgG1, IgG2a and IgG2b. Results are expressed as mean +/− S.E.M. n = 5 mice, * P<0.05, ** P<0.01 (Fishers LSD, Hochberg correction).

*Itk*-KD mice have significantly increased plasma levels of IgE, IgG1 and IgG2a, observed in both male and female mice whereas the levels of IgA, IgG2b and IgG3 were equivalent in the plasma of *Itk*-KD and WT mice (Figure 6). Although T cells from the *Itk*-KD mice were shown to be defective in both Th1 and Th2 cytokine production following *in vitro* activation, serum levels of cytokines were measured to see if elevated cytokines *in vivo* might be responsible for the increased antibody production. However, no increase in Th1, Th2 (data not shown) or the B cell survival factors IL-6 (58.7±7.2 and 47.9±4.1 pg/ml) and BAFF (7037±237 and 7158±206 pg/ml) were detected in the plasma of *Itk*-KD mice compared to WT respectively.

### Comparison of γδT cells in the Itk-KD and WT mice

**Figure 7:**
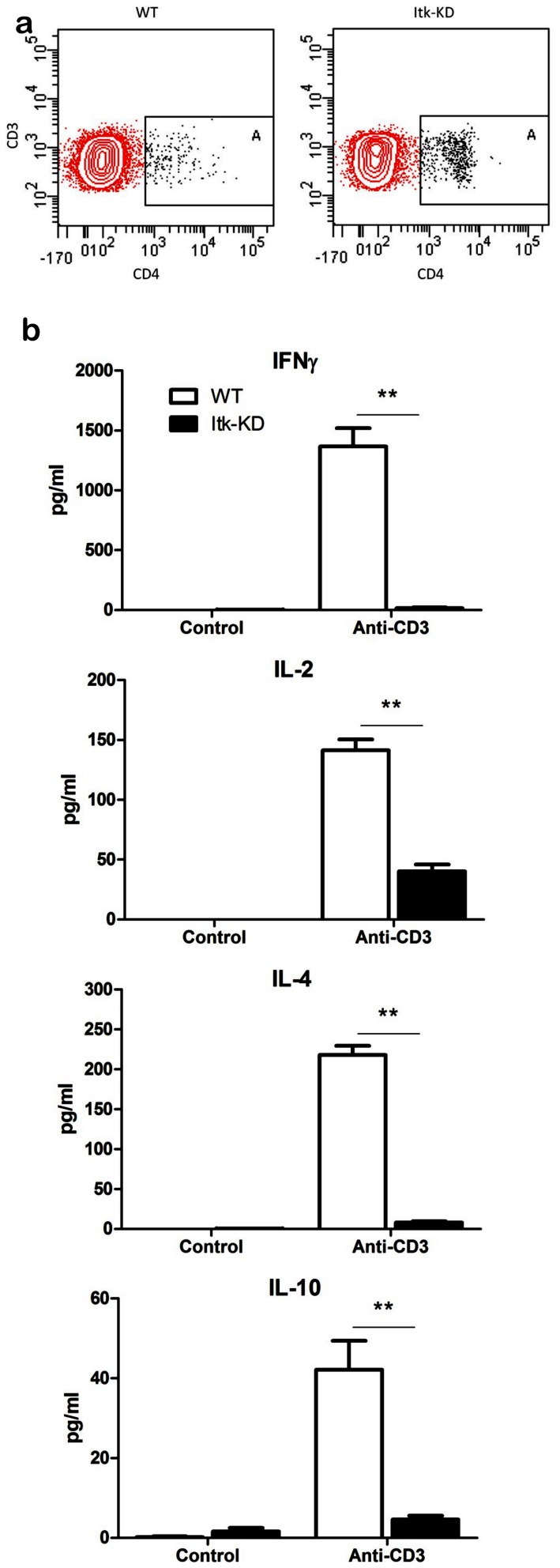
Investigation of γδ cells from the spleens of naïve mice. (a) Purified γδ cells from the spleens of naïve *Itk*-KD mice have an increased percentage of CD4^+^ cells. Spleens from 6 mice per genotype were pooled prior to purification of γδ cells. Following purification, 96% of the live CD3 cells were γδ positive, and of these cells 5.2% were CD4^+^ from *Itk*-KD mice compared to 1.9% for the WT (Box A). (b) Purified γδ cells from *Itk*-KD mice show reduced cytokine release compared to WT controls following *in vitro* activation with anti-CD3. Results are expressed as mean +/− S.E.M. of triplicate cultures of γδ cells from the spleens of 6 mice per genotype.

**Figure 8:**
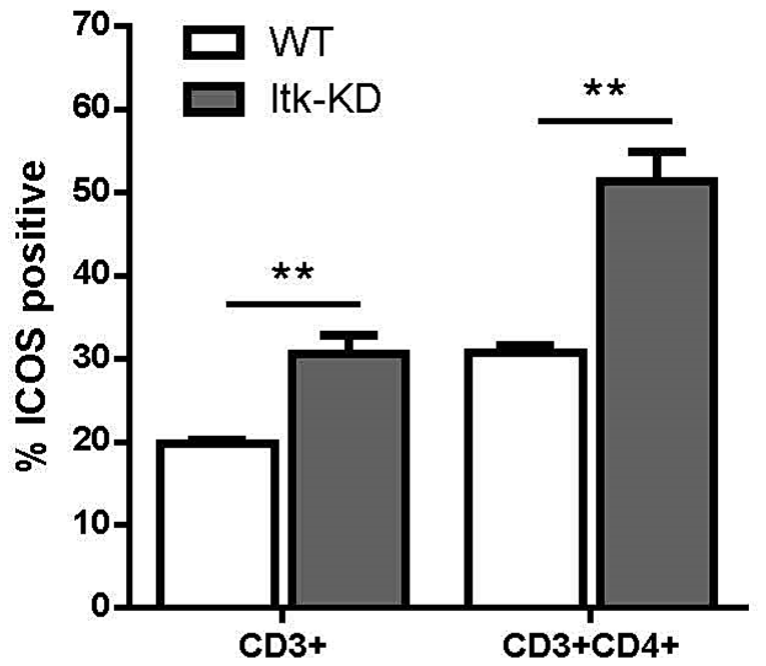
Upregulated expression of ICOS. ICOS expression is upregulated on the surface of CD3^+^ αβ cells in *Itk*-KD splenocytes compared to WT mice. This difference is further enhanced when gating on CD3^+^ CD4^+^ αβ cells. αβ cells were identified as CD3^+^ cells negative for γδ. Results are expressed as mean +/− S.E.M. (n = 6 mice). ** P<0.01 (Fishers LSD, Hochberg correction).

A similar IgE phenotype was observed in previous studies of *Itk* knock out mice, and an increased proportion of CD4^+^ γδT cells, with an aberrant phenotype was thought to be responsible for the increased IgE levels [20], [21]. In the spleen of naïve mice, the proportion of CD3^+^ cells expressing the γδTCR receptor was very low for both WT (0.9±0.08%, n = 6) and *Itk*-KD mice (1±0.1%, n = 6). However, following purification of CD3^+^ γδ cells from splenocytes, a modest increase in the proportion expressing CD4 was observed in *Itk*-KD (5.2% CD4^+^) compared to WT (1.9% CD4^+^; Figure 7a). Further investigation demonstrated that γδ T cells from WT but not *Itk*-KD mice produced cytokines in response to plate bound anti-CD3 (Figure 7b). The inability of *Itk*-KD γδ T cells to release cytokines was seen for both Th1 (IFNγ) and Th2 (IL-4) cytokines. Analysis by flow cytometry indicated that the cell surface marker ICOS was expressed at similar levels on purified γδ T cells from Itk-KD and WT mice (data not shown). In contrast, ICOS expression was markedly increased on αβCD3^+^ cells from the spleens of naïve *Itk*-KD compared to WT mice and the difference between *Itk*-KD and WT was further enhanced when gating on the αβ CD4^+^ CD3^+^ cells (Figure 8).

### Altered response to OVA-induced airway inflammation in the Itk-KD mice

**Figure 9:**
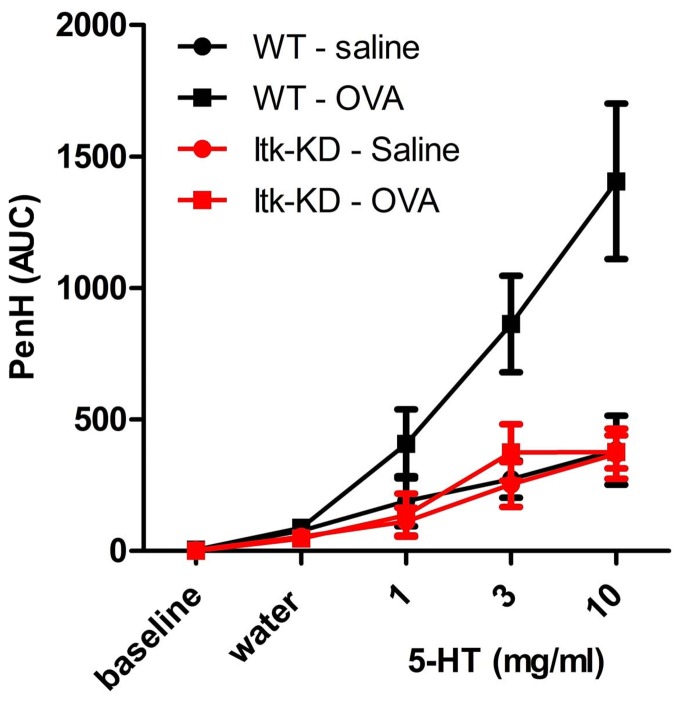
Airway hyper-reactivity (AHR) in OVA model. Female WT and *Itk*-KD mice were sensitised to OVA emulsified in aluminium hydroxide by intraperitoneal injection on day 0 and 14. On day 24, 25 and 26, mice were challenged by the intranasal route with either OVA (WT-OVA and Itk-OVA) or saline (WT-saline or Itk-saline). AHR to a 5HT challenge was assessed on day 27 using whole body plethysmography (PenH). *Itk*-KD mice develop significantly reduced airway hyper-reactivity following sensitisation and challenge to OVA compared to WT (p<0.01, Tukey). Results are expressed as mean+/− S.E.M. (n = 10 for Itk-KD and n = 12 for WT).

WT mice that have been both sensitised intraperitoneally (i.p.) to OVA/alum and challenged intranasally (i.n.) with OVA show a dose–dependent hyper-reactive bronchoconstriction (as measured by PenH) to aerosolised 5-HT. The intranasal OVA challenge is required to produce this hyper-reactivity, as a much reduced response to 5-HT is seen in the WT mice that were only sensitised. In contrast, *Itk*-KD mice that have been sensitised to OVA/alum and challenged with OVA do not develop a hyper-reactive bronchoconstriction to 5-HT (Figure 9). The degree of bronchoconstriction induced by 5-HT in the *Itk*-KD sensititsed and challenged mice is similar to *Itk*-KD mice or WT mice that have only been sensitised.

**Figure 10:**
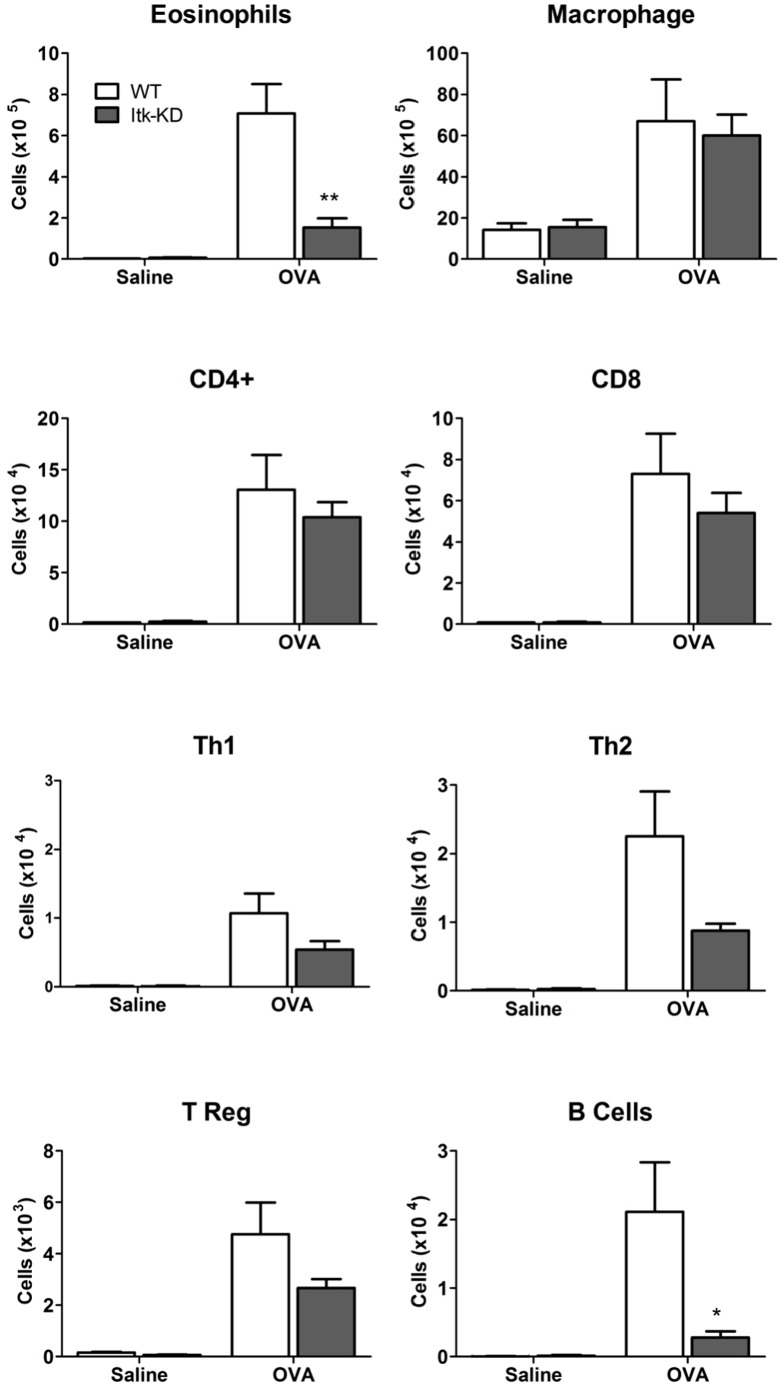
BAL cellular infiltration in OVA model. Cell populations in the BAL of OVA-sensitised *Itk*-KD and WT mice, 48 h after intranasal challenge with OVA or saline. Significant reductions of eosinophils and B cells and non-significant reductions in Th1, Th2 and Treg cells were seen in the BAL of *Itk*-KD mice. Results are expressed as mean +/− S.E.M. (n = 6), * p<0.05, **P<0.01 (Anova with post hoc planned comparison to wild type (un-paired students T test).

**Figure 11:**
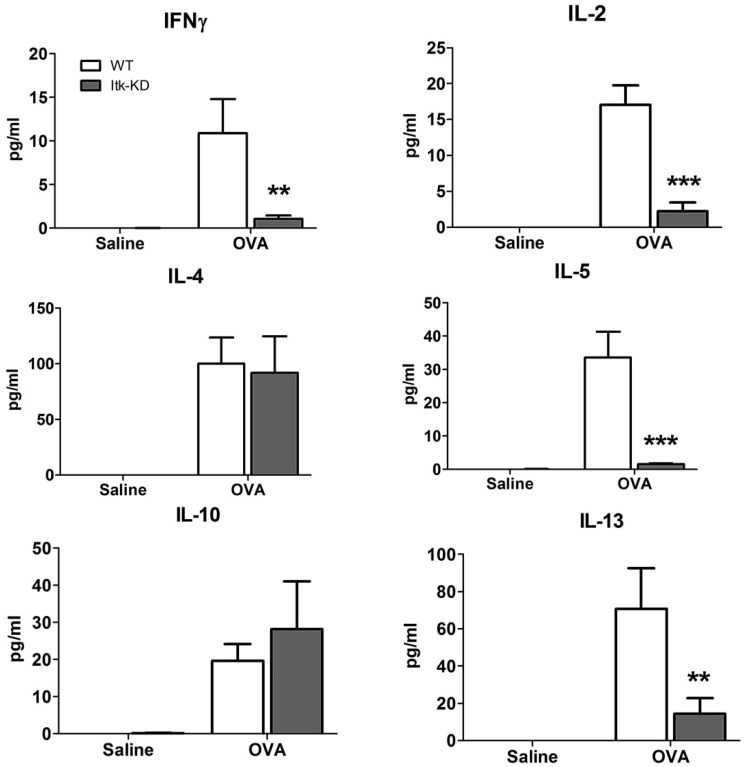
BAL cytokines in OVA model. Cytokine levels in the BAL of OVA-sensitised *Itk*-KD and WT mice, 5 h after intranasal challenge with OVA or saline. Itk-KD mice show reduced levels of IFNγ, IL-2, IL-5 and IL-13 but not IL-4 or IL-10 in the BAL of mice 5 hours after intranasal challenge with OVA. Results are expressed as mean +/− S.E.M. (n = 6), **P<0.01, ***P<0.001 (Tukey).

**Figure 12:**
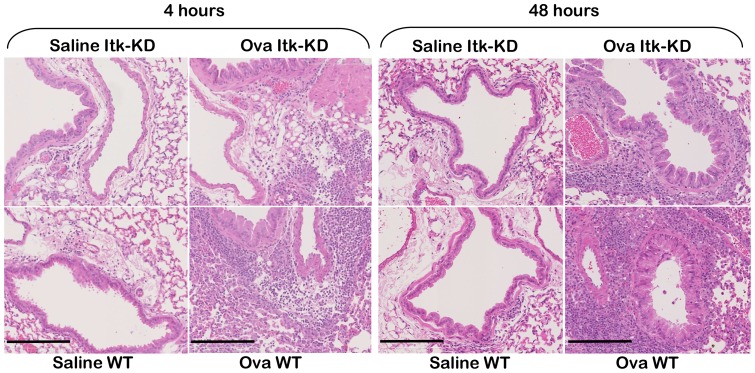
Histopathology of lungs from OVA model. Reduced inflammation was observed in the lung of *Itk*-KD compared to WT mice following sensitisation and challenge with OVA. All animals were sensitised to OVA, and culled 4 hours or 48 hours after intranasal challenge with OVA or saline. Upper panels Itk-KD, lower panels WT. Scale bar  =  200 µm.

In OVA/alum sensitised WT mice a marked influx of eosinophils, macrophages and lymphocytes was measured in the BAL 48 h after the last i.n. challenge with OVA, compared to control mice that were challenged i.n. with saline (Figure 10). The CD4^+^ T lymphocytes were the major lymphocyte population within the BAL following sensitisation and challenge, but a marked CD8^+^ infiltrate was also present, with a lesser proportion of B cells. There was a greater predominance of Th2 over Th1 CD4^+^ cells, as expected given the sensitisation protocol and the BALB/c background of the mice. In comparison with the WT mice, *Itk*-KD mice showed significantly reduced eosinophil and B cell counts in the BAL, but not CD4^+^, CD8^+^ T cells or macrophages (Figure 10). Further analysis of the CD4 population showed a reduction in the Th1, Th2 and Treg populations in the Itk-KD mice compared to the WT sensitised and challenged controls, but this reduction did not reach significance. In sensitised and challenged WT mice there was a substantial increase in cytokine levels in the BAL 4 h after the final OVA challenge compared to sensitised WT mice that were challenged with saline (Figure 11). By contrast, the levels of IFNγ, IL-2, IL-5 and IL-13 were significantly attenuated in *Itk*-KD mice following OVA challenge. However, levels of IL-4 and IL-10 were equivalent in the BAL of *Itk*-KD and WT following the sensitisation and challenge. Cytokine levels were below the limit of detection in the BAL of either WT or *Itk*-KD control mice that were sensitised but challenged with saline. Histological analysis of lung sections showed reduced inflammation in sensitised and challenged *Itk*-KD mice compared to WT mice (Figure 12) although significant accumulation of eosinophils and T cells could still be detected in the lung parenchyma (Figure S4 in File S1)

Analysis of the lymphocyte populations within the mediastinal lymph nodes of sensitised and challenged mice by flow cytometry demonstrated a reduced presence of B cells and CD4^+^ T cells in the *Itk*-KD mice compared to WT controls whereas the CD8^+^ T cells were equivalent between the two genotypes (data not shown). Analysis of the mediastinal lymph nodes indicated that the gross morphology of the *Itk*-KD lymph nodes was similar to WT (data not shown).

**Figure 13:**
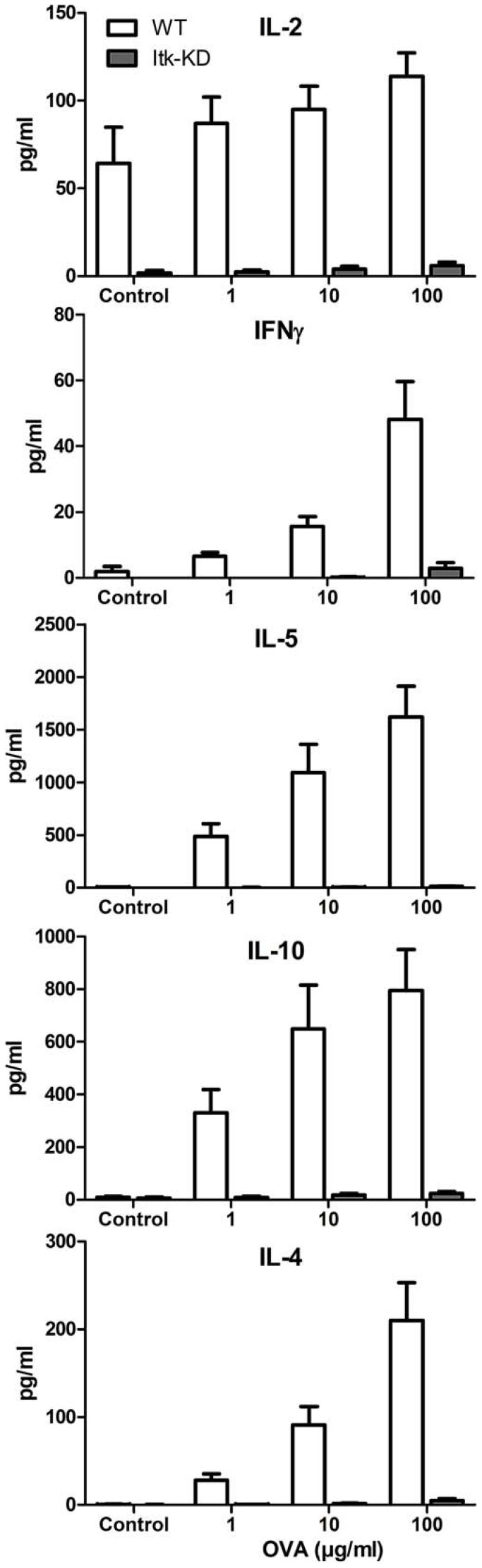
Reactivation of mediastinal lymph node cells from OVA model. Following sensitisation and challenge of mice with OVA, mediastinal lymph node cells from WT and *Itk*-KD mice were reactivated *in vitro* with OVA for 72 hours. In WT mice this induces a concentration-dependent increase in cytokine release, measured in culture supernatants. However, under the same conditions mediastinal lymph node cells from *Itk*-KD mice fail to secrete cytokines. Results are shown as mean +/− S.E.M. (n = 12 mice).

Following sensitisation and challenge of mice with OVA, the *in vitro* reactivation of mediastinal lymph node cells from WT mice with OVA for 72 hours induced a concentration-dependent increase in cytokine (IFNγ, IL-2, IL-10, IL-4 and IL-5) release in culture supernatants. However, under the same conditions mediastinal lymph node cells from *Itk*-KD mice failed to secrete cytokines (Figure 13). Similar results were seen following reactivation of spleen cells with OVA, whereby cells from WT but not *Itk*-KD mice could release cytokines (Figure S5 in File S1). The failure to secrete cytokines is not believed to be due to a deficiency in CD4^+^ cells as intracellular cytokine staining following activation with PMA/ionomycin revealed an increased percentage of IFNγ and IL-4 producing CD4^+^ cells present in both the lymph node and spleen of *Itk*-KD mice compared to WT controls (Figure S6 in File S1). Furthermore, flow cytometry analysis of the spleen, indicated that CD4^+^ cells were present at a similar percentage in both *Itk*-KD and WT mice, although a reduction in CD4^+^ cells was observed in the lymph node.

### Altered antibody response to OVA in Itk-KD mice

**Figure 14:**
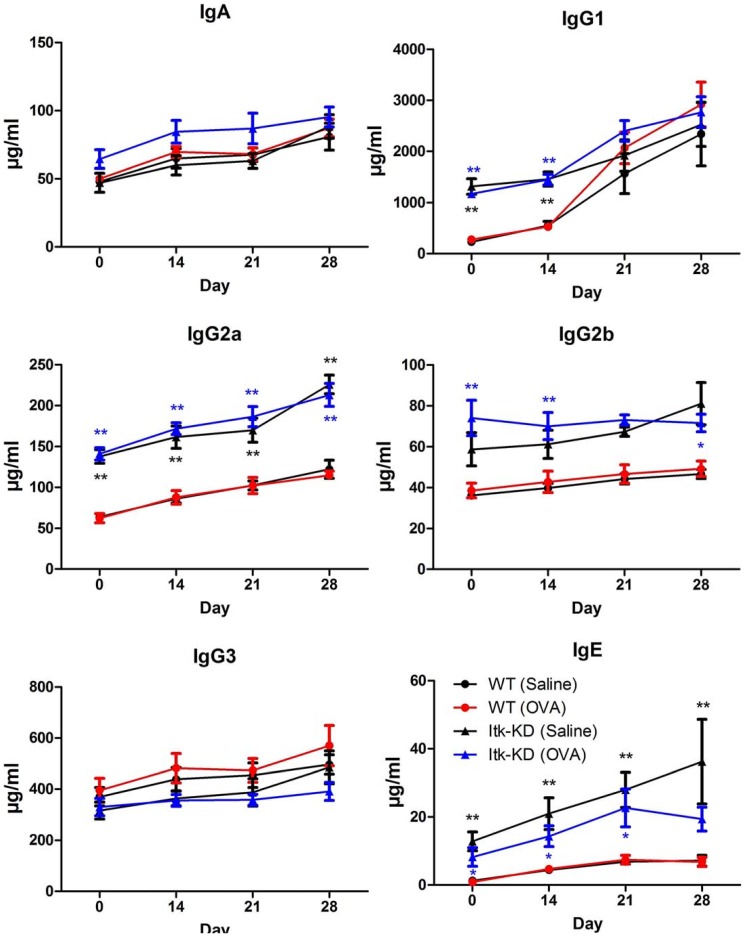
Antibody levels in the serum from OVA model. Serum immunoglobulin levels were determined in WT and *Itk*-KD mice following sensitisation with OVA and challenge with either saline (Saline) or OVA (OVA). Results are expressed as mean +/− S.E.M.. (n = 5 mice for saline control, n = 6 mice for OVA challenge) * P<0.05, **P<0.01 compared to the WT control (significance between the OVA groups is shown in blue and WT in black; Fishers LSD, Hochberg correction).

Following on from observations of deregulated antibody production in naïve *Itk*-KD mice, we investigated the antibody response over the time course of the OVA/alum sensitisation and OVA challenge (Figure 14). WT mice showed an increase in the antibody titres in response to OVA/alum sensitisation, the most marked increase was for IgG1, with IgG2b and IgG3 remaining relatively constant over the time-course. *Itk*-KD mice had elevated levels of IgE, IgG1, IgG2a and IgG2b in the pre-bleed samples compared to WT controls, in agreement with the data from naïve mice as described above. However, antibody titres of IgE, IgG2a and IgG2b were significantly greater in *Itk*-KD mice than WT controls over the duration of the sensitisation/challenge time course. In contrast, titres of IgG1 increase markedly between day 14 and 21 in the WT, such that by day 21 titres were equivalent between the WT and *Itk*-KD mice. Levels of IgA and IgG3 were similar in the pre-bleeds of WT and Itk-KD mice, and there was no significant difference between the two genotypes over the duration of the time-course. *Itk*-KD mice retained the ability to generate OVA specific IgE as there was no significant difference between the levels of OVA-specific IgE in the terminal bleeds of Itk-KD and WT mice (Figure S7 in File S1).

## Discussion

Numerous previous studies have identified ITK as an important component of the TCR signalling complex and evidence from knockout mice have suggested it plays a particularly central role in Th2 function, so implicating it as a drug target for allergic diseases. ITK is not absolutely required for the development of T-lymphocytes, as T cells are present in *Itk* ^−/−^ mice, but these mice have a reduced number of CD4^+^ cells, with an altered CD4/CD8 balance [16]. The CD4 and CD8 cells both have an altered phenotype, with reduction in cells expressing a naïve phenotype and an increase in cells with an innate, memory phenotype [22]. Peripheral naïve CD4^+^ cells from *Itk* ^−/−^ mice appear to have increased expression of Th1 signature genes including Eomesdermin, IFNγ, T-bet and IL12RβI and the enhanced IFNγ expression is thought to feed forward to suppress the Th2 phenotype since removal of IFNγ rescues the Th2 phenotype [23].

*In vitro* activation of CD4^+^ cells from *Itk* ^−/−^ mice show reduced levels of IL-2 [16], [18], selective reduction in Th2 [17] or both Th1 and Th2 cytokines compared to wild type [19]. Naïve T cells from *Itk* ^−/−^ mice can differentiate normally into either Th1 or Th2 cells if cultured *in vitro* under appropriate cytokine conditions, suggesting that ITK is not required for Th2 cell differentiation [19], [24], [25]. However, studies differ in the reported effect of ITK knockout on cytokine release upon re-stimulation, showing either a selective reduction in Th2 [24] or reduction in both Th1 and Th2 cytokines [19], . This difference may be due to the genetic background of the mice or the culture conditions.

Most studies to date suggest ITK plays a particularly important role for the development of a Th2 response. *Itk* ^−/−^ mice fail to clear *Nippostrongylus brasiliensis* and *Schistosoma mansoni* infection due to a failure to mount an effective Th2 response [17], [19]. Further evidence for defective Th2 immunity is that *Itk* ^−/−^ mice are resistant to developing airway inflammation and hyperreactivity in response to ovalbumin (OVA) sensitisation and challenge [26]–[28]. *Ex-vivo* activation of CD4^+^ cells isolated from draining lymph nodes of OVA sensitised mice showed complete inhibition of IL-5 and IL-13, but partial reduction in IFNγ and IL-10 [26]. *Itk* ^−/−^ mice show reduced levels of IL-4 and IL-13 but not IFNγ (mRNA and protein) as measured in the lungs of *Itk* knockout mice compared to wild type mice (WT) [27], [28] and this effect was accompanied by a significant reduction in the Th2 promoting transcription factor GATA3 [27]. Furthermore, *Itk* ^−/−^ mice showed a reduced response in a model of atopic dermatitis [29]. In human studies, genetic analysis has shown SNPs in *Itk* are associated with atopy, allergic rhinitis and asthma [30], [31].

We have chosen to replace the *Itk* gene with a kinase dead allele since it has been shown that knock out of ITK disrupts multiple components of the TCR signalling complex, showing that a knockout is unlikely to be an accurate translational model for exploring the therapeutic potential of a small molecule ITK kinase inhibitor. Mutation of K390R has been shown to inactivate the kinase activity of ITK [2], [5] and we have successfully generated a transgenic mouse replacing the wild type *Itk* allele with this kinase dead mutation. The BALB/c background was chosen as this strain is routinely used for the OVA model of airway inflammation in many laboratories. Historically the majority of gene targeted mouse models were generated using 129 ES cells so using BALB/c not only reduced the number of animals necessary to fully backcross onto the desired strain but also the length of time to achieve production of study populations.

We confirmed that the ITK-K390R is expressed in CD4^+^ cells and has an identical molecular mass to WT ITK. It was also important to demonstrate that ITK from the K390R kinase dead mouse retained scaffold function, in particular an ability to couple to actin polymerisation since T cells from *Itk* ^−/−^ mice show reduced actin polymerisation following activation of TCR/CD3 [10]. Reduction in ITK expression in Jurkat cells or primary T cells by small interfering mRNA disrupts TCR-induced actin polymerisation, which could be restored by transfection with WT or kinase inactive (K390R) murine ITK, but not by ITK constructs carrying mutations in PH or SH2 domains [6]. It is known that actin polymerisation is required for full TCR signalling [32] therefore since the scaffold function of ITK and not the kinase function plays an important role in actin polymerisation in response to TCR activation, it was important to demonstrate that T-cells from our *Itk*-KD mice retained an ability to polymerise actin in response to TCR stimulation and we suggest that the interpretation of T-cell trafficking defects in *Itk* ^−/−^ mice need to be treated with caution [33].

Multiple studies have demonstrated that ITK is essential for the secretion of IL-2 and Th2 cytokines following activation of T cells via the CD3/TCR complex [16]–[19], [24], [25]. We have confirmed these findings and demonstrated that splenocytes from *Itk*-KD mice have a broadly attenuated cytokine release in response to soluble anti-CD3. Therefore the attenuation of cytokine release from anti-CD3 activated splenocytes from *Itk*-KD mice compared to WT is fully consistent with the ITK-K390R being devoid of kinase activity. This severely impaired T cell derived cytokine response to activation with anti-CD3 cannot be explained in terms of a reduced proportion of CD4^+^ cells, as we did not observe any gross changes in the CD4^+^ cell numbers in the spleens of *Itk*-KD compared to WT mice. CD4^+^ cells from *Itk*-KD mice show an increased percentage of cells producing IFNγ and IL-4 in response to PMA/ionomycin stimulation, confirming that the lack of cytokine release in response to anti-CD3 is a deficiency in the signalling pathway between CD3 and the generation of DAG and Ca^++^ second messengers. It has been reported that *Itk* ^−/−^ mice have greatly decreased numbers of CD4^+^ cells with the naïve CD62L^high^ CD44^low^ phenotype but no effect on the number of CD62^low^ CD44^high^ innate memory phenotype, leading to an increased ratio of innate memory to naïve CD4^+^ cells [22]. These innate memory phenotype CD4^+^ cells carry preformed message for IFNγ [22]. *Itk*-KD mice have an increase in the proportion of CD4^+^ cells that express the memory markers CD62L^low^ CD44^high^ in comparison to WT controls, whilst the proportion of CD62L^high^ CD44^low^ naïve phenotype was reduced, suggesting that the increased proportion of CD62L^low^CD44^high^ cells may be responsible for the increased proportion of cells expressing intracellular IFNγ. On an *Itk* ^−/−^ background, expression of ITK in a T cell dependent manner (under CD2 promoter) can partially rescue the reduction in CD62L^high^ CD44^low^ naïve cells, whereas expression of mutant ITK lacking its kinase domain had no effect [22]. Therefore the results from the *Itk*-KD mice support that ITK kinase activity is required for the normal development of naïve CD4^+^ cells.

T cells play an important role in immunoglobulin class switching, both directly via co-stimulatory molecule expression, and indirectly via cytokine secretion. As *Itk* ^−/−^ mice show decreased T cell cytokine production, it is perhaps surprising that elevated IgE has been reported in naïve *Itk* ^−/−^ [19] with an increased percentage of B cells in the spleen of *Itk* ^−/−^ mice which have undergone class switch to express IgE [4]. Therefore, we have extended these observations to investigate the levels of IgE and other antibody isotypes in the serum of *Itk*-KD mice. As previously reported in *Itk* ^−/−^ mice, the naïve *Itk*-KD mice have significantly increased plasma levels of IgE and also IgG1 compared to WT mice. Switching to IgE and IgG1 is regulated by IL-4 [34], [35]. Although *Itk*-KD mice showed deficient IL-4 release in response to *in vitro* stimulation via antigen or anti-CD3, levels of IL-4 in the BAL were equivalent from WT and *Itk*-KD mice following *in vivo* OVA sensitisation and challenge, suggesting that *Itk*-KD mice can generate IL-4 under pro-inflammatory conditions, perhaps from a non-T cell. However, increased antibody levels are not restricted to the IL-4 dependent isotypes, as *Itk*-KD mice also express increased levels of IgG2a, which is dependent on the Th1 cytokine, IFNγ [36], [37]. Therefore, *Itk*-KD mice have severely attenuated ability to secrete T cell cytokines but enhanced switching to IgE, IgG1 and IgG2a which are promoted by the T cell cytokines IL-4 and IFNγ, perhaps suggesting an alternative cell source for these cytokines in the absence of functional ITK. *Itk* ^−/−^ αβ NKT cells are also defective in production of IL-4 [38], but studies have indicated that the γδ subset of T cells are aberrant in *Itk* ^−/−^ mice and may secrete increased levels of IL-4 [20], [21]. The γδ T cells have been shown to modulate the humoral response, not only through cytokine release but also B cell co-stimulatory molecule expression and induction of germinal centres. In *Itk* ^−/−^ mice it has been demonstrated that the γδ T cells play an essential role in the elevated IgE, as normal IgE levels are observed when the *Itk* ^−/−^ mice are crossed with mice lacking the TCR δ chain [20]. Furthermore, *Itk* ^−/−^ mice have increased numbers of CD4 γδ T cells [20], [21]. In contrast to *Itk* ^−/−^ αβ T cells, when *Itk* ^−/−^ γδT cells are activated via the γδTCR they secrete significant amounts of Th2 cytokines and up-regulate co-stimulatory molecules including ICOS and CD40L [20], [21]. However the role of γδ cells in the increased antibody production in *Itk*-KD mice is less clear. In the spleen of naïve mice, the proportion of CD3^+^ cells expressing γδ was low, at approximately 1% for both WT and *Itk*-KD, whilst the proportion of CD4^+^ γδ cells is increased in *Itk*-KD compared to WT, which is in agreement with the *Itk* ^−/−^ studies [20], [21]. However, ICOS expression was similar on *Itk*-KD and WT γδ cells, and they are deficient in both Th1 and Th2 cytokine production following activation via anti-CD3. Therefore the *Itk*-KD are different in this respect to *Itk* ^−/−^ mice and it is unlikely that γδ cells are responsible for the aberrant antibody response. These differences to the studies using *Itk* ^−/−^ mice may be attributed to the absence of ITK scaffold function in the *Itk* ^−/−^ mice, but may also be influenced by the genetic background of the mice (BALB/c for the Itk-KD mice compared to C57BL/10 or C57BL/6 for the *Itk* ^−/−^ mice), and also the differences in methodology to isolate and activate the γδ cells.

In contrast to the normal ICOS expression on *Itk*-KD γδ cells, ICOS is significantly up-regulated on *Itk*-KD αβ cells, which is particularly marked in the CD3^+^CD4^+^ cells. ICOS is not normally expressed on resting cells and yet in naïve *Itk*-KD mice approximately 50% of the CD3^+^CD4^+^ cells are positive for ICOS. Therefore, the aberrant antibody production seen in the *Itk*-KD mice may, at least in part, be due to the increased expression of ICOS on αβ cells which are the dominant TCR type. ICOS^−/−^ mice exhibit a profound deficit in Ig class switching and impaired germinal centre formation [39], [40]. In naïve ICOS^−/−^ mice, levels of IgM were normal, indicating the B cell responses are not directly dependent on ICOS [39], however there was a marked reduction in IgG1, IgG2a and IgE, and in *Itk*-KD mice which have enhanced ICOS expression these antibodies are increased. ICOS^−/−^ mice show a modest increase in IgG3 [39], although there may be a trend for reduced IgG3 in the *Itk*-KD, there is no significant effect. Therefore the data from ICOS^−/−^ mice is consistent with the hypothesis that it is the enhanced ICOS expression on αβ T cells in *Itk*-KD mice that results in the abnormal antibody profile, and possibly for the enhanced germinal centres reported in the spleens of the male mice. In contrast, CD40L expression on naïve splenic CD4^+^ cells was comparable between WT and *Itk*-KD mice (data not shown).

*Itk*-KD mice were protected from OVA-induced airway inflammation which is broadly in agreement with similar studies in the *Itk* ^−/−^ mice [26]–[28]. However, there are some differences observed between the *Itk*-KD and earlier *Itk* ^−/−^ studies. In the *Itk*-KD mice, there is no evidence to suggest that inhibition of ITK will selectively inhibit Th2 whilst sparing Th1 cytokines, as has been reported for *Itk* ^−/−^ mice [27], [28]. This difference may not be solely attributed to the genetic background of the mice as *Itk* ^−/−^ on a BALB/c background have also been reported to significantly reduce IL-4 production in the absence of a significant effect on IFNγ [17], however this was following infection with *L.major*. We and others [22] have shown that T cells deficient in ITK activity contain elevated levels of IFNγ within the T cell, but that this is not released following activation via CD3/TCR activation. However, *Itk* ^−/−^ mice can secrete IFNγ in response to innate stimuli, such as IL-12/IL-18 [22], perhaps this may explain the apparent Th1 sparing reported in some infection studies. Indeed, inhibition of both Th1 and Th2 cytokines was observed with ITK inhibitors in human PBMC and lung [12].

Previous studies in *Itk* ^−/−^ have demonstrated a decrease in IL-4, IL-5 and IL-13 in the BAL [28], [41] following sensitisation and challenge with OVA. In the *Itk*-KD mice there was also a significant inhibition of IFNγ, IL-2, IL-5 and IL-13 in the BAL from mice that have been sensitised and challenged with OVA compared to WT mice. However, levels of IL-4 in the BAL of *Itk*-KD mice are comparable to the WT. The *ex vivo* restimulation with OVA of splenocytes or lymph node cells from *Itk*-KD fail to secrete T cell cytokines including IL-4, confirming that *Itk*-KD T cells are unable to produce IL-4 in response to TCR stimulation. However, intracellular cytokine staining following activation with PMA/ionomycin, thereby by-passing ITK, indicates that lymph node or spleen of *Itk*-KD mice contain the same number of IL-4^+^ cells, therefore in the absence of ITK kinase activity T cells can differentiate normally into Th2 or Th1, but cannot produce cytokines in response to TCR activation. This is in agreement with earlier studies using *Itk* ^−/−^ mice. The cellular source of IL-4 in the BAL has not been defined, but eosinophils are significantly reduced in both the BAL and in the lung tissue, suggesting they are not the source of the IL-4. Although the γδ T cell constitutes a minor population of T cells in the blood and peripheral organs, they may be substantially increased in the mucosal epithelia. However, in the lungs of *Itk*-KD mice following OVA sensitisation and challenge, the percentage of CD3^+^ cells expressing the γδ TCR was low (<2%, data not shown) and similar between *Itk*-KD and WT. This finding, together with the functional data demonstrating reduced cytokine secretion, including IL-4, suggests that γδ T cells are unlikely to be the source of the IL-4 measured in the BAL. Therefore it is interesting that IL-4 can be generated locally in the tissues at the site of inflammation in the absence of ITK kinase activity, this IL-4 may support the switching to IgE in the presence of enhanced ICOS stimulation.

Chemokines control the migration of lymphocytes to the site of inflammation, and ITK plays a role in the CXCL12/CXCR4 and CCL11/CCR3 mediated migration and adhesion by altering the actin cytoskeleton [28], [42]. Furthermore, *Itk* ^−/−^ mice show significant reduction in CD4^+^ cells in the BAL following sensitisation and challenge to OVA [26], [28], [41]. In the *Itk*-KD mice, the influx of T cells was reduced, and this was evident when looking at the lung sections and in the BAL, but this was not the complete inhibition reported in the *Itk* ^−/−^ studies described above. This may suggest that the scaffold function of ITK plays a role in the regulation of actin polymerisation in response to CXCR4 and CCR3 activation, as reported for following TCR activation. However, this has not been directly addressed in this study and a previous study demonstrated actin polymerisation in response to CCL11/CCR3 activation was indeed dependent on the kinase activity of ITK, using a transgenic mouse where the kinase domain is replaced by green-fluorescent protein under the Lck promoter, on a WT or *Itk* ^−/−^ background [28].

*Itk* ^−/−^ and *Itk*-KD mice show a marked increase in total IgE, and some interesting studies demonstrated that mast cells from *Itk* ^−/−^ mice fail to degranulate to antigen, as the mast cell FcεRI receptors are saturated with polyclonal IgE [4]. This may suggest that reduced airway inflammation and airway hyperresponsiveness may, in part be due to reduced degranulation of mast cells. However, the OVA-model of airway inflammation is predominantly a T cell dependent model. Studies in mast cell-deficient (W/W^v^) mice demonstrate that mast cells do not play a role in the inflammatory response to OVA, as measured by cellular influx and cytokine levels in the BAL, but do play a role in the AHR [43]. Furthermore, airway inflammation and hyperreactivity is transferred by CD4^+^ cells from WT mice following OVA sensitisation and challenge into *Itk* ^−/−^ recipients, indicating that the OVA model is indeed T cell dependent [27].

In addition to a potential role in asthma, ITK may play a role in other allergic diseases and the pathogenesis of inflammatory skin diseases. Single nucleotide polymorphisms, in the 5′ region of the *Itk* genomic locus have been associated with atopy and eczema [30]. ITK mRNA levels in the peripheral blood T cells is elevated in atopic dermatitis patients compared to healthy controls, and this increase correlates with disease severity [44]. Furthermore, ITK expression is increased in lesional skin from patients with atopic dermatitis and allergic contact dermatitis [29]. *Itk* ^−/−^ mice show significantly reduced inflammation in models of contact hypersensitivity, and this anti-inflammatory effect was reproduced by systemic administration of an ITK inhibitor [29].

The *Itk*-KD mice show many similarities to *Itk* ^−/−^ mice, but also some differences. The data presented with the *Itk*-KD mice supports the rationale for the use of ITK-inhibitors for the treatment of T cell-mediated diseases, however, it also highlights a potential for ITK inhibition to result in a distortion of the normal antibody response, and identifies upregulation of ICOS on αβ cells as a possible cause of this aberrant response. However, as in these mice the ITK is rendered kinase dead constitutively, this may be a developmental impact of ITK inhibition and not associated with inhibition of ITK once the immune system has developed. Therefore it is recommended that antibody levels and ICOS expression be carefully monitored following long term administration of selective ITK inhibitors in any future toxicology or clinical studies.

## Supporting Information

File S1 **Supplementary Figures.**
(DOCX)
