# Supplementary material for: Characterisation of a K390R ITK Kinase Dead Transgenic Mouse – Implications for ITK as a Therapeutic Target
Source: PLoS One. 2014 Sep 24;9(9):e107490. doi: 10.1371/journal.pone.0107490 (PMC4174519; doi:10.1371/journal.pone.0107490)
Supplement: File S1 — Supplementary Figures. (DOCX) [file pone.0107490.s001.docx]

**File S1: SUPPLEMENTARY INFORMATION**

**File S1: Supplementary Figure S1**


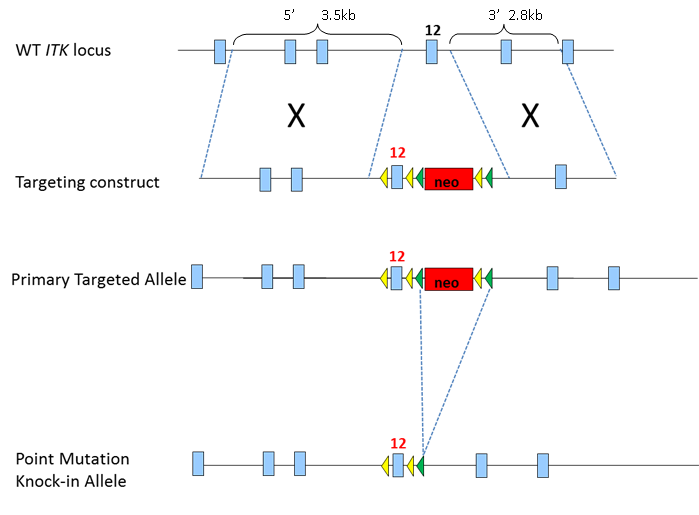


**File S1: Supplementary Figure S1**. The *Itk* targeting strategy

A targeting strategy was devised to introduce the point mutation K390R into exon 12 of the *Itk* gene by homologous recombination in ES cells. 5’ & 3’ homology arms (approx. 3.5 & 2.8kb respectively) flanking exon 12 were generated using Phusion High-Fidelity DNA Polymerase (New England BioLabs) on a BALB/c genomic DNA template. Similarly a ~0.6kb fragment carrying exon 12 lying between these two homology arms was isolated and subjected to site-directed mutagenesis with the QuickChangeII site-directed mutagenesis kit (Stratagene) to introduce the appropriate point mutation (A to G mutation at n1169 of the cDNA sequence). The 5’ & 3’ homology arms and the mutated exon 12 fragments were subcloned into a parental targeting vector to achieve the positioning of the loxP (green arrowhead) & FRT sites (yellow arrowheads) and the neo cassette as indicated. Gene targeting was performed in de novo generated BALB/c ES cells. The targeting construct was linearised and electroporated into ES cells according to standard methods. ES cells correctly targeted at the 3’end were identified by Southern blot analysis using a PCR-derived external probe. Correct gene targeting at the 5’ end and the presence of the appropriate point mutation was confirmed by sequencing of a ~6kb PCR product. Note that an additional loxP site was simultaneously introduced into intron 11 , but was not used in subsequent model generation. Targeted ES cell clones were injected into C57Bl6/J-derived blastocysts, and resultant male chimaeras were crossed with BALB/c females to produce mice heterozygous for the *Itk* primary targeted allele. These were subsequently bred to a germline Flp-deleter strain resulting in mice heterozygous for the *Itk* knock in allele. After an expansion breed to BALB/c study populations were produced by intercrossing sufficient heterozygous pairs to produce homozygote and wild type litter mates.

**File S1: Figure S2**


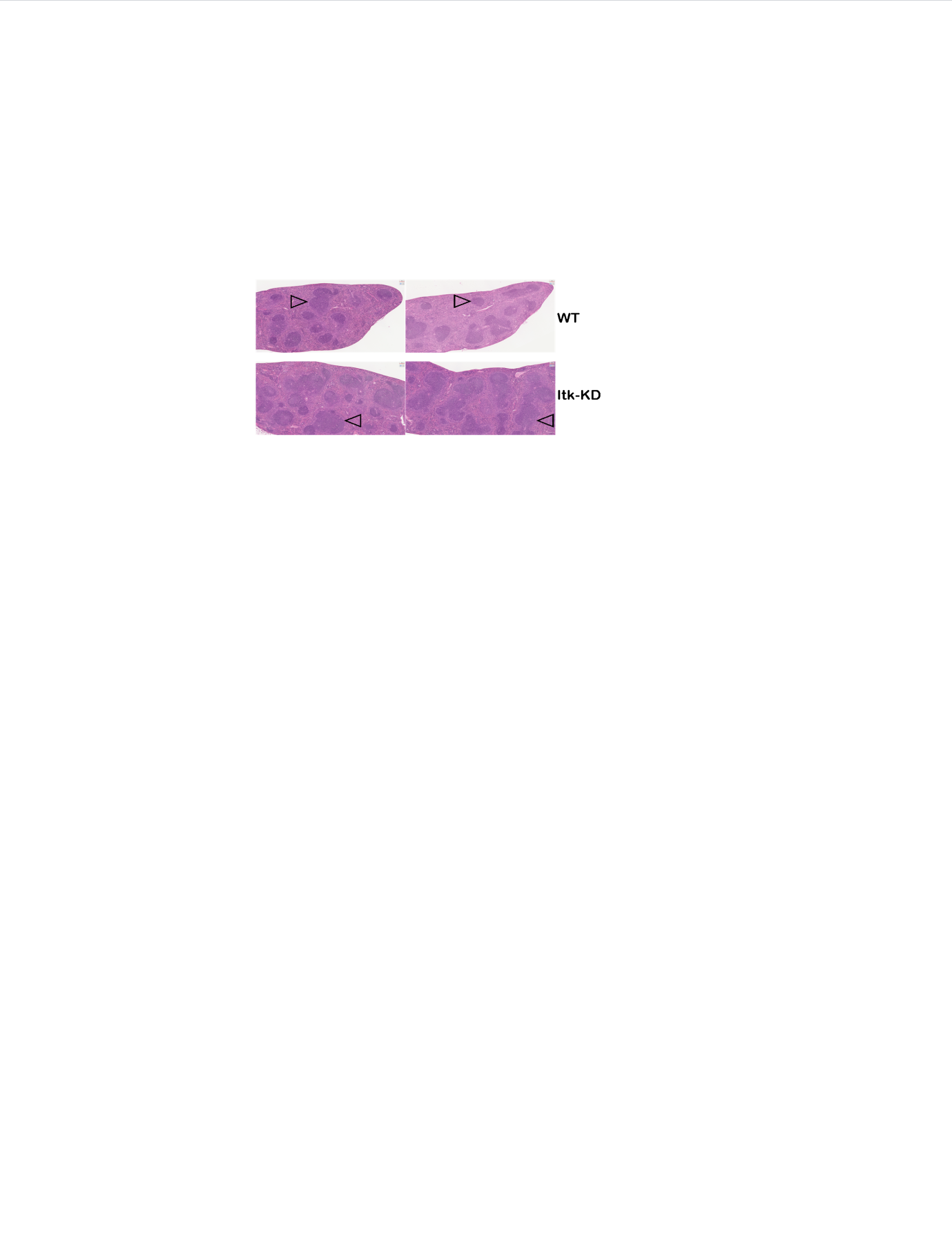
**File S1: Figure S2**. Increased germinal centres in the spleens of *Itk*-KD mice. Spleens were removed from WT and *Itk*-KD mice, fixed and processed for Haematoxylin and Eosin staining as described in Materials and Methods. Two representative spleens from WT and *Itk*-KD mice are shown for comparison. Germinal centres are shown by arrowheads.

**File S1: Supplementary Figure S3A**


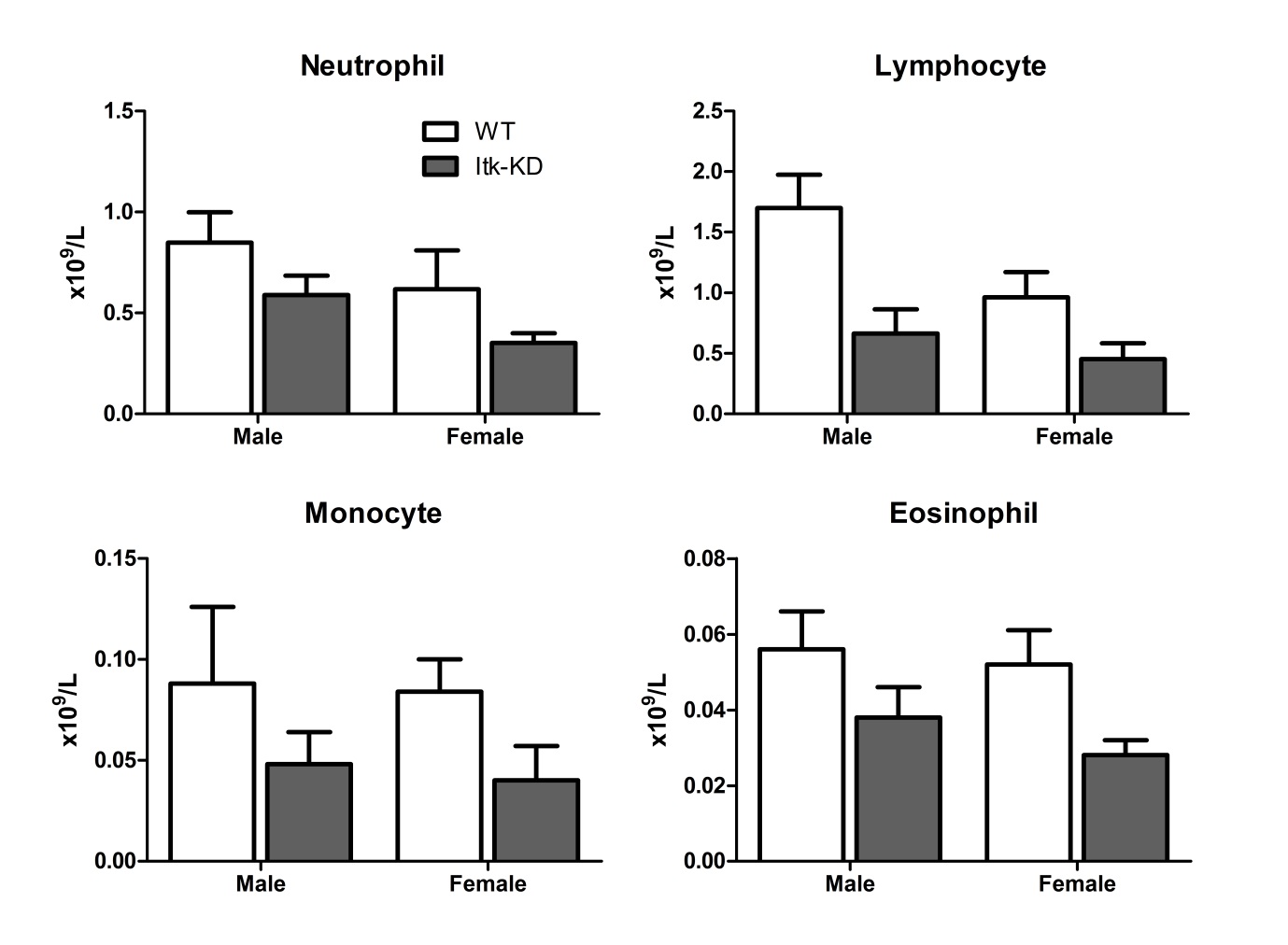


**File S1: Supplementary Figure S3A.** Absolute counts for neutrophils, lymphocytes, monocytes and eosinophils were measured in the blood of naive mice using ADVIA 120. Results are the means +/- S.E.M. of n=5 mice.

**File S1: Supplementary Figure S3B**


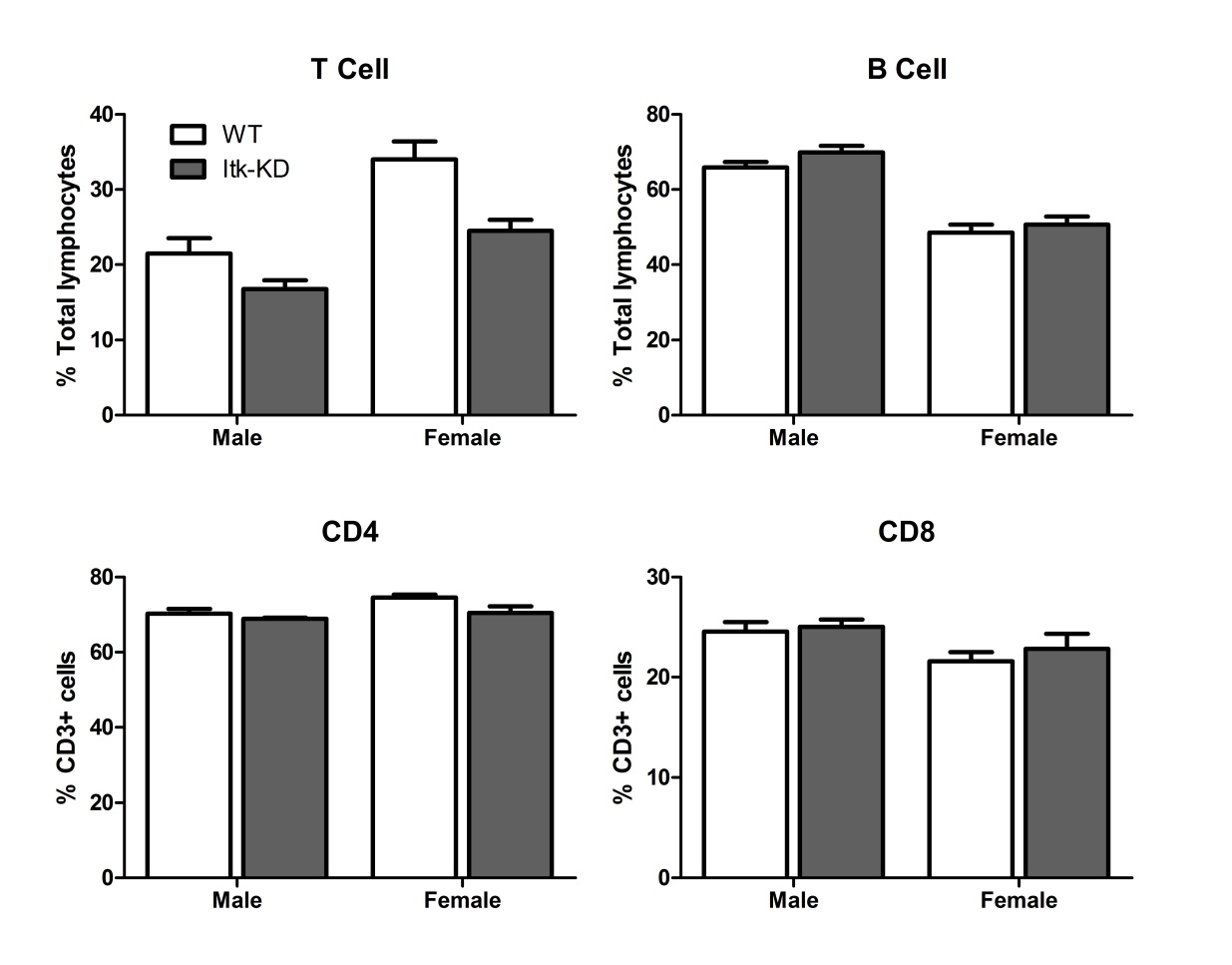


**File S1: Supplementary Figure S3B.** Further analysis of the lymphocytes in the blood was carried out by flow cytometry. The percentage of T cell (CD3^+^) and B cell (CD19^+^) of total lymphocytes (identified by CD45 and FSC/SSC profile). The CD3^+^ population was further analysed as CD4 and CD8 positive cells. Results are the means +/- S.E.M. of n=5 mice.

Each endpoint was analysed independently using analysis of variance. Where data was not normally distributed a log10 transformation was applied. Each analysis included the factors of genotype (WT vs. *Itk*-KD) and gender as explanatory variables, as well as the interaction between genotype and gender. Analysis of the interaction demonstrates whether the difference in the responses between WT and *Itk*-KD is constant between the two sexes. No significant interaction was seen; therefore significance is reported as the difference between WT and Itk-KD, irrespective of gender. Due to multiple comparisons a more robust P<0.01 was taken as significant.

*Itk*-KD mice have significantly reduced absolute lymphocyte count (p=0.0027) and percentage of T cells (p=0.0009).

**File S1: Supplementary Figure S4**

B Eosinophils

Saline Itk-KD Ova Itk-KD

Saline WT Ova WT


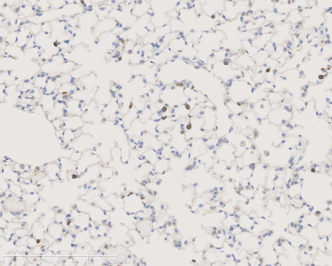

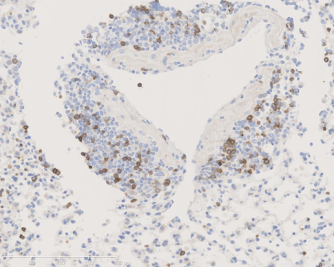

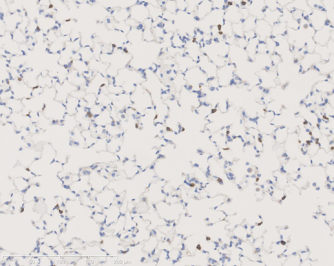

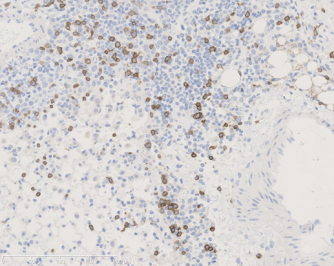


Saline Itk-KD Ova Itk-KD

Saline WT Ova WT

A T-cells

**File S1: Supplementary Figure S4.** Histology of lungs from OVA model. Mice were sensitised to OVA, and culled 4 hours after intranasal challenge with OVA or saline. Upper panels *Itk*-KD, lower panels WT. OVA challenge induces T cell (A) and eosinophil (B) infiltration into the lungs compared to saline in both *Itk*-KD and WT mice. T cells were identified by anti-CD3 staining and eosinophils by anti-major basic protein. Histological analysis of the cell infiltration by eye using a 5-point scoring system indicated a modest reduction in the T cell and a reduction in eosinophil infiltration in the *Itk*-KD compared to WT control (data not shown). All assessments were made without knowledge of the mouse genotypes. Scale bar = 200µm.

**File S1: Supplementary Figure S5**


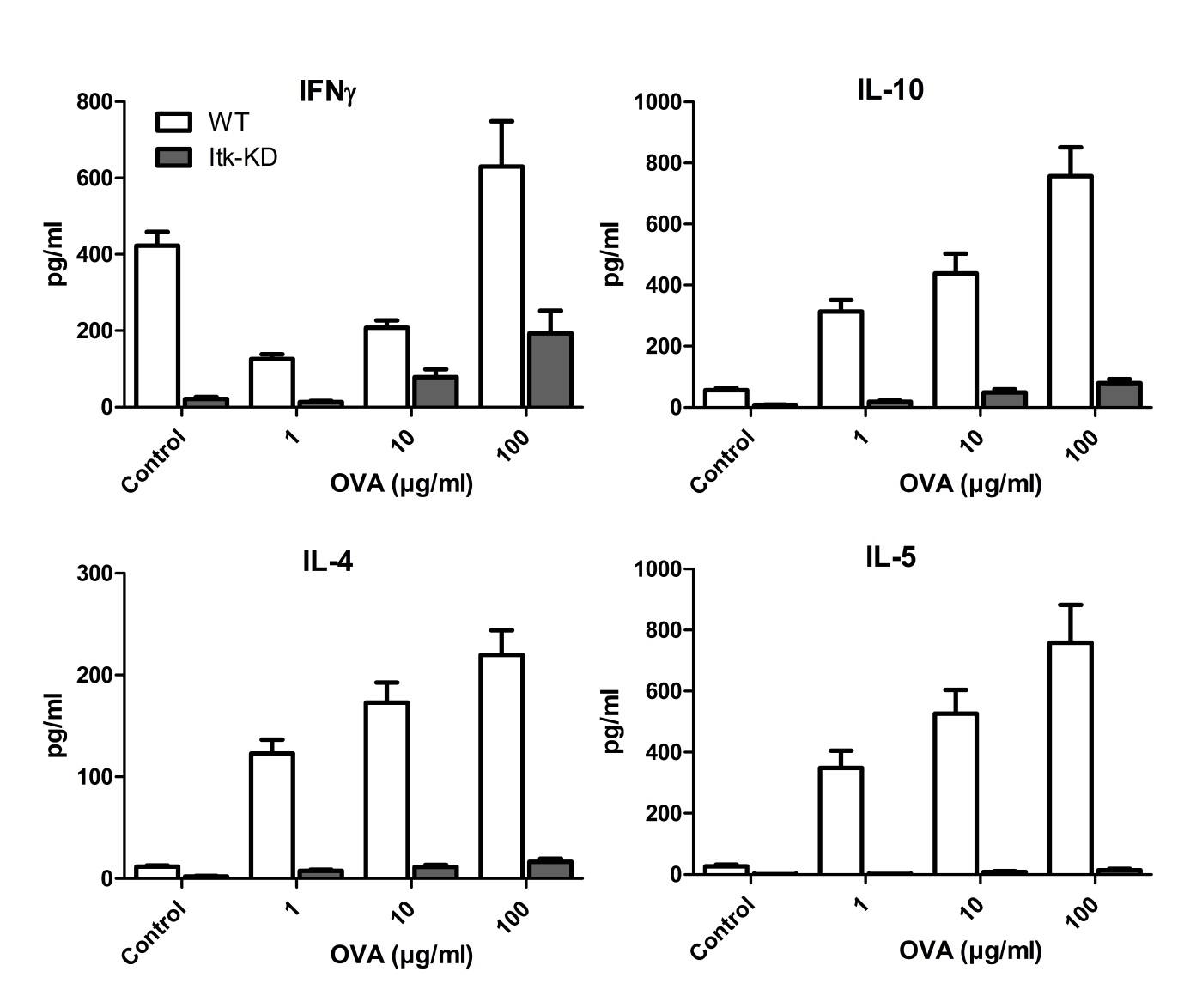


**File S1: Supplementary Figure S5.** Following sensitisation and challenge of mice with OVA, the *in vitro* reactivation of splenocytes from WT mice with OVA induces a concentration-dependent increase in cytokine (IFNγ, IL-10, IL-4 and IL-5) release, measured in culture supernatants following a 72h incubation. However, under the same conditions splenocytes from *Itk*-KD mice fail to secrete cytokines. Results are the means +/- S.E.M. from n=12 mice.

**File S1: Supplementary Figure S6**


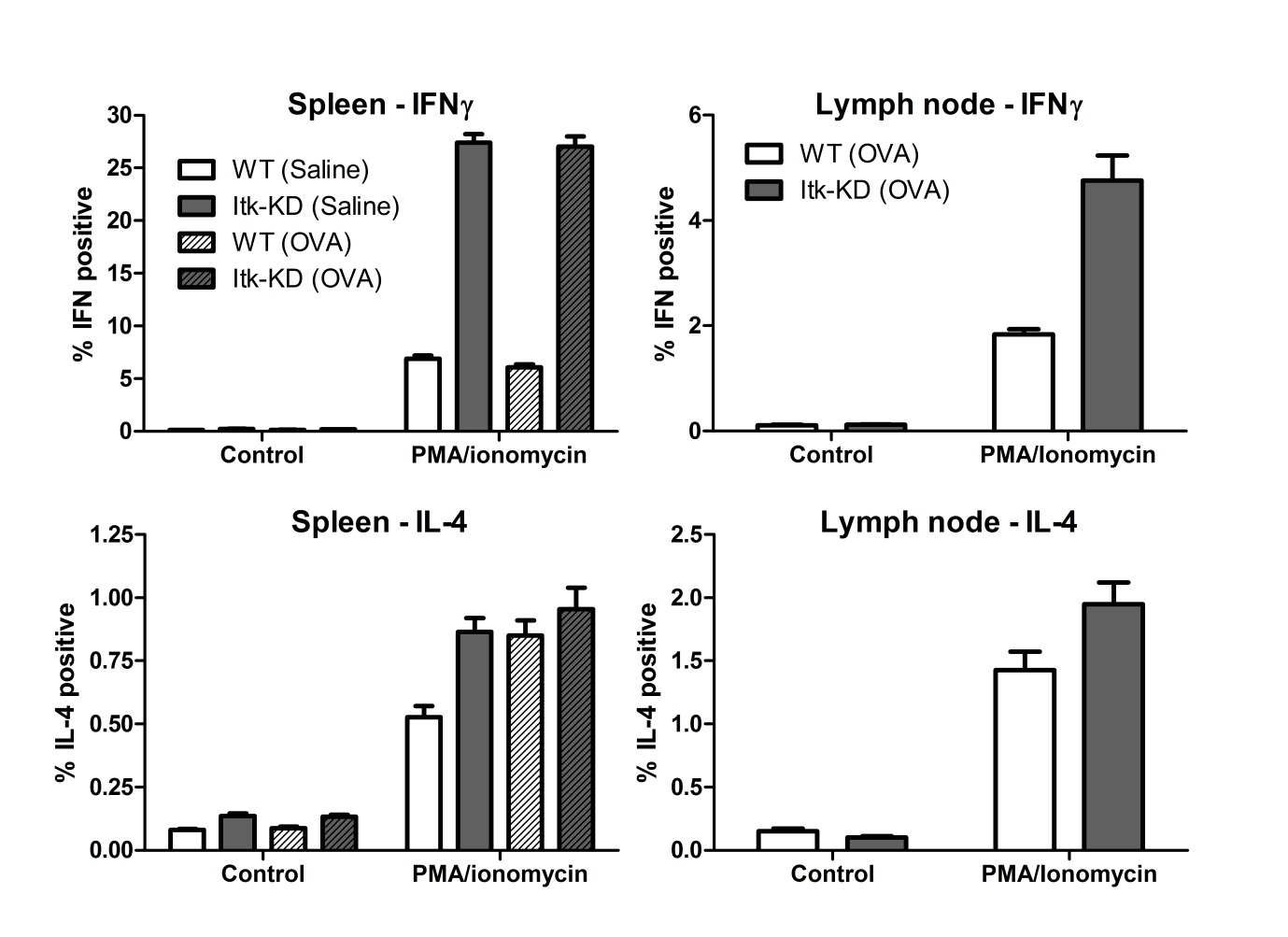


**File S1: Supplementary Figure S6.** Following sensitisation and challenge of mice with OVA, CD4^+^ cells present in both the lymph node and spleen of *Itk*-KD mice and WT controls were reactivated *in vitro* with PMA /ionomycin and intracellular cytokine staining revealed an increased percentage of IFNγ and IL-4 producing CD4^+^ cells present in both the lymph node and spleen of *Itk*-KD mice compared to WT controls. Results are the means +/- S.E.M. from n=12 mice.

**File S1: Supplementary Figure S7**


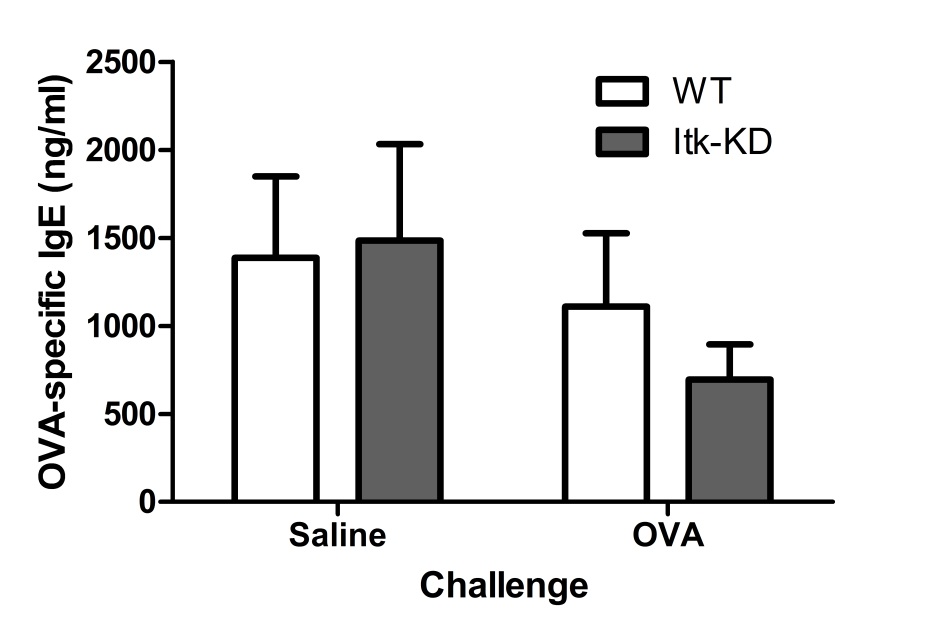


**File S1: Supplementary Figure S7.** OVA specific IgE antibody production in the OVA/alum sensitisation and OVA challenge model. *Itk*-KD mice retain the ability to generate OVA specific IgE and there is no significant difference between the levels of OVA-specific IgE in the terminal bleeds of Itk-KD and WT mice. Results are the means +/- S.E.M. from n=5 mice. There is no significant difference between the WT and *Itk*-KD groups.
